# Microglia jointly degrade fibrillar alpha-synuclein cargo by distribution through tunneling nanotubes

**DOI:** 10.1016/j.cell.2021.09.007

**Published:** 2021-09-30

**Authors:** Hannah Scheiblich, Cira Dansokho, Dilek Mercan, Susanne V. Schmidt, Luc Bousset, Lena Wischhof, Frederik Eikens, Alexandru Odainic, Jasper Spitzer, Angelika Griep, Stephanie Schwartz, Daniele Bano, Eicke Latz, Ronald Melki, Michael T. Heneka

**Affiliations:** 1 Department of Neurodegenerative Disease and Geriatric Psychiatry/Neurology, University of Bonn Medical Center, 53127 Bonn, Germany; 2 German Center for Neurodegenerative Diseases (DZNE), 53127 Bonn, Germany; 3 Institute of Innate Immunity, University of Bonn Medical Center, 53127 Bonn, Germany; 4 Institut François Jacob, MIRCen, CEA and Laboratory of Neurodegenerative Diseases, CNRS, 92265 Fontenay-aux-Roses, France; 5 Divison of Infectious Diseases and Immunology, University of Massachusetts Medical School, 01605 Worcester, MA, USA

**Keywords:** microglia, alpha-synuclein, tunneling nanotubes, cell-to-cell transfer, clearance, LRRK2, synucleinopathies, degradation

## Abstract

Microglia are the CNS resident immune cells that react to misfolded proteins through pattern recognition receptor ligation and activation of inflammatory pathways. Here, we studied how microglia handle and cope with α-synuclein (α-syn) fibrils and their clearance. We found that microglia exposed to α-syn establish a cellular network through the formation of F-actin-dependent intercellular connections, which transfer α-syn from overloaded microglia to neighboring naive microglia where the α-syn cargo got rapidly and effectively degraded. Lowering the α-syn burden attenuated the inflammatory profile of microglia and improved their survival. This degradation strategy was compromised in cells carrying the LRRK2 G2019S mutation. We confirmed the intercellular transfer of α-syn assemblies in microglia using organotypic slice cultures, 2-photon microscopy, and neuropathology of patients. Together, these data identify a mechanism by which microglia create an “on-demand” functional network in order to improve pathogenic α-syn clearance.

## Highlights

• Microglia rapidly engulf exogenous α-synuclein but hesitate in its degradation
• α-synuclein is transferred between microglia through tunneling nanotubes
• Healthy microglia donate mitochondria to α-synuclein overloaded cells
• Sharing the α-synuclein burden attenuated the inflammatory microglia profile

## Graphical abstract

**Figure:**
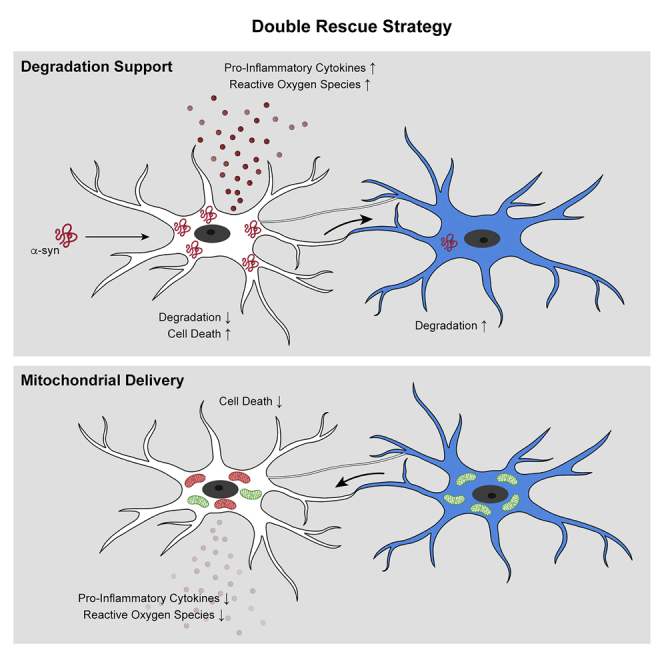

## Teaser

Microglia form F-actin dependent intercellular networks to transfer α-synuclein fibrils to neighboring microglial cells for degradation and clearance. Impairment in this process, as seen with Parkinson’s disease mutations, leads to increased inflammatory profiles and cell death.

## Introduction

Several synucleinopathies including Parkinson’s disease (PD) and dementia with Lewy bodies (DLB) are characterized by the presence of intraneuronal cytoplasmic inclusions called Lewy bodies (LB) that are rich in an aggregated form of the protein α-synuclein (α-syn) (Spillantini et al., 1997). α-syn is a 14 kDa protein with no defined structure (Weinreb et al., 1996) that is primarily produced in neurons. Under pathological conditions, the monomeric form of the protein progressively forms oligomeric structures and insoluble fibrillar assemblies that, together with crowded organellar components (Shahmoradian et al., 2019), accumulate in LBs. Overexpression of α-syn or mutations in the *SNCA* gene that encodes for α-syn cause progressive locomotor deficits and loss of dopaminergic neurons in the *substantia nigra* (Blesa and Przedborski, 2014).

Of note, recent evidence suggests that α-syn pathology may spread by cell-to-cell transmission, thereby contributing to disease progression (Desplats et al., 2009; Hansen et al., 2011; Lee et al., 2010; Rostami et al., 2017). Several mechanisms including exocytosis and endocytosis, uptake of exosomes carrying α-syn, or direct penetration may account for such cell-to-cell transmission (Emmanouilidou et al., 2010; Flavin et al., 2017; Freundt et al., 2012; Masuda-Suzukake et al., 2013) and may be limited by efficient microglial identification and clearance (Choi et al., 2020). Therefore, increasing the clearance of α-syn and lowering the accompanying protein accumulation may be a promising therapeutic strategy for the treatment of synucleinopathies.

Being the brain’s primary innate immune cells, microglia play a crucial role in mediating cerebral homeostasis by sensing changes in their immediate environment, clearing cellular debris, and providing neurotrophins (Colonna and Butovsky, 2017). Upon ligation of pattern recognition receptors (PRRs), microglia become activated and execute an inflammatory response that, in case it persists, causes chronic neuroinflammation and neuronal damage (Colonna and Butovsky, 2017; Heneka et al., 2014). Evidence for such a chronic neuroinflammatory response can be found in brains of PD patients and other synucleinopathies, where microglial activation occurs in all brain regions where aggregated α-syn accumulates (Croisier et al., 2005; Gerhard et al., 2006; McGeer et al., 1988). It has been hypothesized that inflammation can promote α-syn aggregation and amplify PD pathology (Brundin et al., 2008) that might lead to a compromised protein clearance by microglia. Excessive effort has been made toward the identification of cellular pathways regulating α-syn clearance in microglia. Nonetheless, the exact mechanism for the clearance of α-syn remains unclear.

Here, we examined how the microglial cell population as a whole deals with the clearance of α-syn fibrils and whether microglial survival is affected upon exposure to fibrillar α-syn. We provide evidence that microglia form an “on-demand” functional network enabling them to share the burden of aggregated α-syn degradation. Lowering the load of α-syn aggregates attenuated the inflammatory profile and cytotoxicity in α-syn-containing microglia by the donation of intact mitochondria. This protective strategy was compromised in microglia carrying the LRRK2 G2019S mutation. Monocyte-derived microglia-like cells (MDMis) that were donated from DLB/PD patients showed an impaired potential to transfer α-syn aggregates compared to their healthy spouses. Thus, our data uncover a strategy for effective aggregated α-syn clearance and prove its existence in microglia *in vitro*, *ex vivo*, and *in vivo*.

## Results

### Fibrillar α-syn induces inflammation and apoptosis in microglia

**Figure S1:**
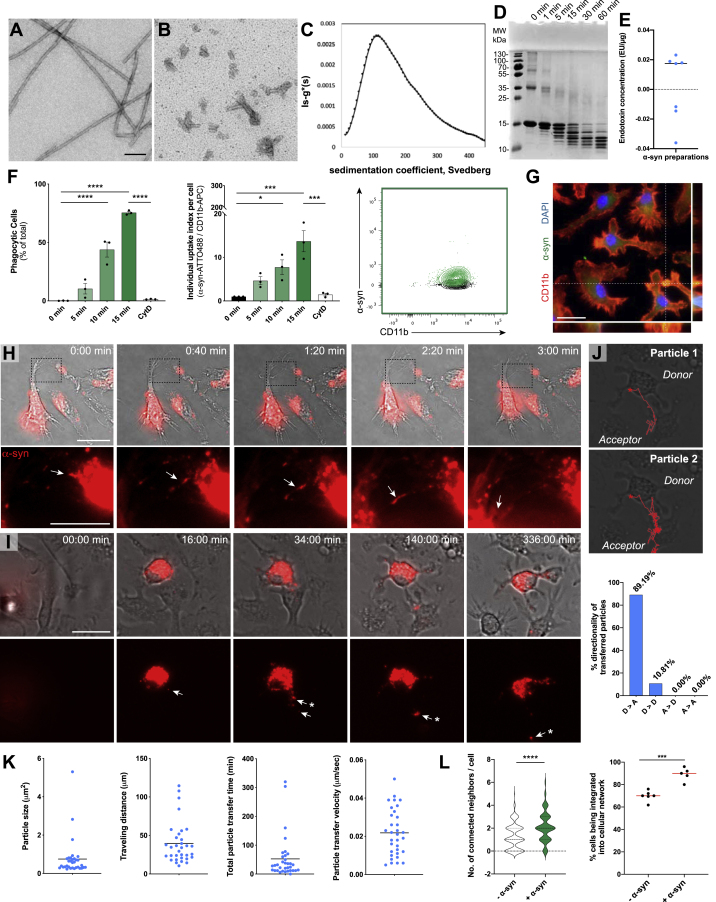
Characterization of α-syn fibrils and its transfer between microglia, related to Figures 1, 2, and 3 (A-B) Characterization of α-syn fibrils used throughout this study. Electron micrographs of α-syn fibrils stained by Uranyl acetate, before (A) and after (B) fragmentation. (C) Sedimentation coefficient of fragmented fibrils determined by analytical ultracentrifugation. The sedimentation velocity measurement shows a distribution centered at 110 Svedberg. The sedimentation coefficient is compatible with a molecular species of 16,000 kDa corresponding to ∼1,100 monomers of 14.46 kDa. (D) Proteolytic profile of fibrillar α-syn. Time course of fibrillar α-syn (100 μM) degradation by proteinase K (0.38 μg/mL) analyzed by SDS-PAGE after Coomassie blue staining. (E) All α-syn preparations were confirmed to have an endotoxin concentration below 0.02 endotoxin units/μg (EU/μg). n = 7 independent α-syn preparations. (F) Quantification of the percentage of α-syn monomers containing microglia (left) and the relative individual uptake index per cell (middle) after exposure to ATTO488-labeled α-syn monomers, 2 μM; n = 3 independent experiments per group. Diagram representing the α-syn monomers uptake as measured by FACS (right). (G) Representative immunostaining showing the internalization of ATTO488-labeled α-syn monomers into CD11b-labeled microglia. (H) Representative time-lapse recordings demonstrating that α-syn fibrils are transferred from one microglia to another via thin cellular membrane connections. (I) Representative time-lapse recording demonstrating that α-syn fibrils are transferred from overloaded microglia to naive microglia via cellular connections. (J) Representative particle tracking of aggregates transferred from donors to acceptors as shown in (I) (upper panels). Quantification of the directionality of transferred particles. D = donors, A = acceptors. A total of 37 particle transfer events were analyzed. (K) Quantification of particles that underwent transfer from α-syn-containing microglia toward naive cells for their size, traveling distance, total particle transfer time, and particle transfer velocity. n = 33 individual particles. (L) Quantification of the number of individual cell neighbors and proportion of cells involved in a network before and after α-syn fibrils uptake. Network formation was analyzed using a CellProfiler script, identifying individual cells and measuring the number of adjacent cells. A total of at least 205 cells per condition were analyzed. n = 5-6 individual experiments. Graphs in F are presented as mean ± SEM and were analyzed by one-way ANOVA followed by Tukey’s multiple comparison post hoc test. Graphs in K present individual particles and the mean. Graphs in L were analyzed by t test analysis. ^∗∗∗∗^p < 0.0001, ^∗∗∗^p < 0.001, ^∗^p < 0.05 compared to 0 min. Scale bars: 100 nm (A-B), 20 μm (G, I), 10 μm (H).

Aggregated α-syn is predominantly found in neurons; however, it also appears frequently in glial cells while disease progresses. To investigate whether and how the microglial cell population as whole deals with the clearance of pathogenic α-syn, we exposed microglia to well-characterized recombinant human α-syn (Figures S1A–S1E). Unless otherwise stated, all following experiments were performed using α-syn fibrils.

**Figure S2:**
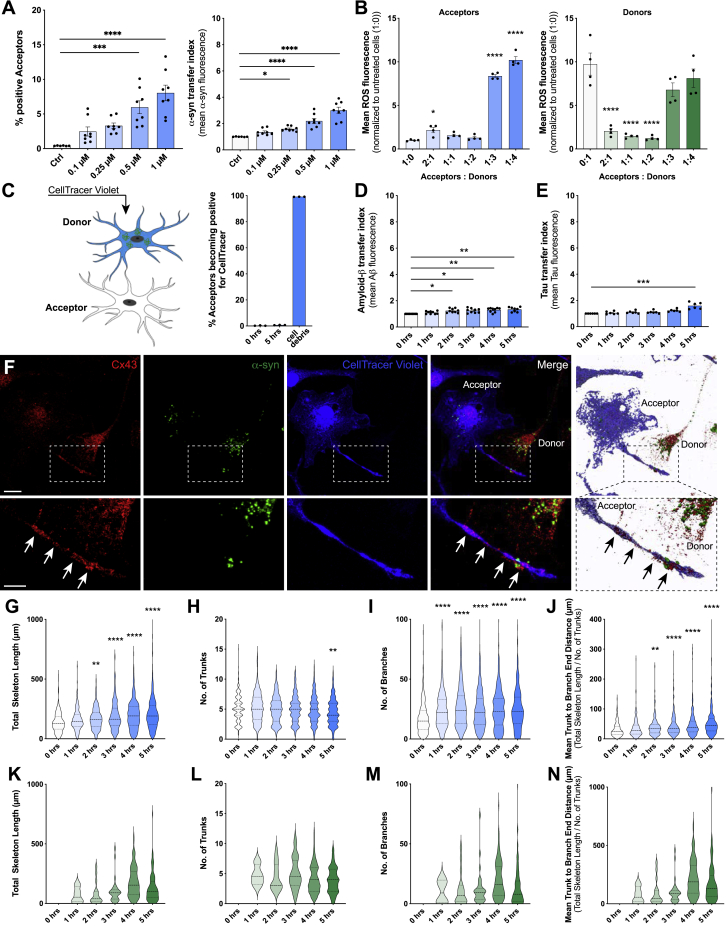
Cell-to-cell contact favors fibrillar α-syn transfer between microglia and induces cytoskeletal changes, related to Figure 3 (A) Dose-response curve analysis for α-syn transfer capacities from donors to acceptors at increasing concentrations (0.1 – 1 μM). n = 4 with duplicate treatments for all conditions. (B) Quantification of the ROS release of donor (right) and acceptor (left) cells with changing donor: acceptor ratios. (C) Schematic drawing of staining strategy and quantification of the rate of acceptors engulfing dying donors (CellTracer labeled). The schematic was created using BioRender.com and Adobe Photoshop. (D) Quantification of the transfer rate of fibrillar Amyloid-β between microglia. n = 3 with triplicate treatments for all conditions. (E) Quantification of the transfer rate of fibrillar Tau between microglia. n = 3 with duplicate treatments for all conditions. (F) Donors (CellTracer negative) and acceptors (CellTracer positive, blue) were co-cultured for 5 h and immunocytochemical analysis for Connexin 43 (Cx43) were performed. (G) Donors and acceptors were co-cultured for the indicated time and the total length of the F-actin cytoskeleton of acceptors was measured. (H–J) Quantification of the number of trunks (H), branches (I) and the mean trunk to branch end distance (J) of acceptor microglia over time. n = 4 per group. A total of at least 185-400 cells were analyzed. (K) Donors and acceptors were co-cultured for the indicated time and the total length of the F-actin cytoskeleton of donors was measured. (L–N) Quantification of the number of trunks (L), branches (M) and the mean trunk to branch end distance (N) of donor microglia over time. n = 4 per group. A total of at least 185-400 cells were analyzed. Graphs in A-E are presented as mean ± SEM and were analyzed by one-way ANOVA followed by Tukey’s multiple comparison post hoc test. Graphs in G-N are presented as violin plots and were analyzed by one-way ANOVA followed by Tukey’s multiple comparison post hoc test. ^∗∗∗∗^p < 0.0001, ^∗∗∗^p < 0.001, ^∗∗^p < 0.01 compared to 0 h. Scale bars: 5 μm.

**Figure S3:**
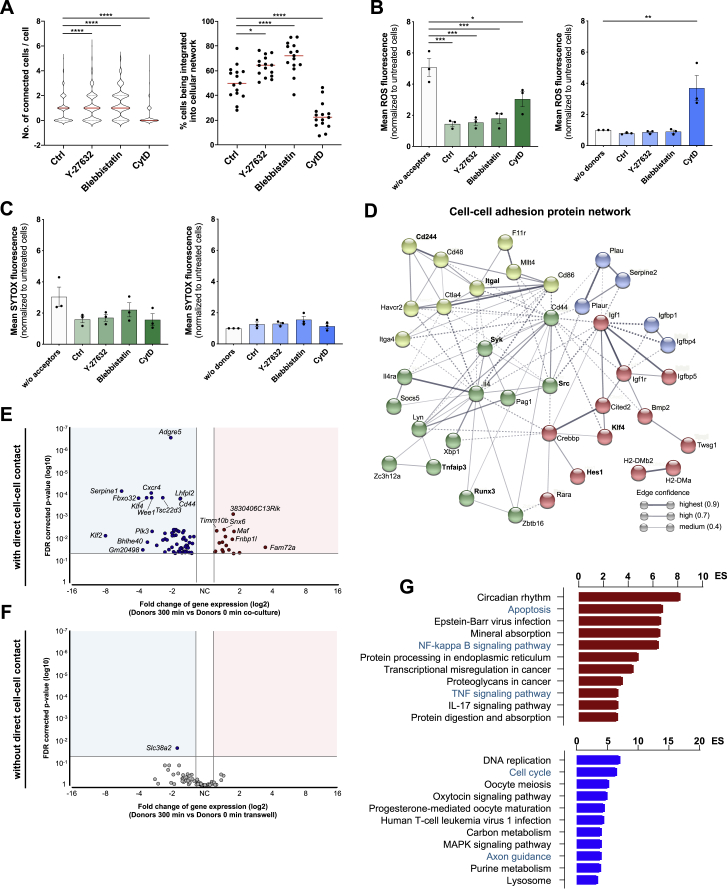
Effects of the cytoskeleton on α-syn transmission and transcriptomic analysis of microglia, related to Figures 3 and 4 (A) Quantification of the number of neighboring cells per individual cell and the percentage of cells being integrated into a cellular network. Network formation was analyzed using a CellProfiler script, identifying individual cells and measuring the number of adjacent cells. A total of at least 815 cells per condition were analyzed. n = 3 with five replicates per group. (B) Quantification of the mean ROS fluorescence of donors (left) and acceptors (right) that were co-cultured for 5 h and treated with Y-27632, Blebbistatin, and CytD, respectively. n = 3 independent experiments. (C) Quantification of the mean SYTOX fluorescence of donors (left) and acceptors (right) that were co-cultured for 5 h and treated with Y-27632, Blebbistatin, and CytD, respectively. n = 3 independent experiments. (D) STRING protein interaction network for 35 proteins associated to cell-cell adhesion based on the differential gene expression between donors and acceptors. Proteins with highest fold changes in expression levels are highlighted in bold. From the top 10 expressed genes, *Sirpb1c* is excluded as it was not connected to the network. A maximum of 10 interactors was allowed. Colors represent the membership to clusters based on k-means clustering. (E) Vulcano plot of genes that were differentially regulated when donors were co-cultured with acceptors with direct cell-cell contact. (F) Vulcano plot of genes that were differentially regulated when donors were co-cultured with acceptors without direct cell-cell contact. (G) Bar chart of most enriched pathways for aggregated α-syn induced (red) and suppressed (blue) genes in donors using the transwell insert strategy. Graphs are presented as mean ± SEM and were analyzed by one-way ANOVA followed by Tukey’s multiple comparison post hoc test (A right, B) or by one-way ANOVA followed by Dunn’s multiple comparison post hoc test (A left). ^∗∗∗∗^p < 0.0001, ^∗∗∗^p < 0.001, ^∗∗^p < 0.01, ^∗^p < 0.05 compared to 0 h.

**Figure 1:**
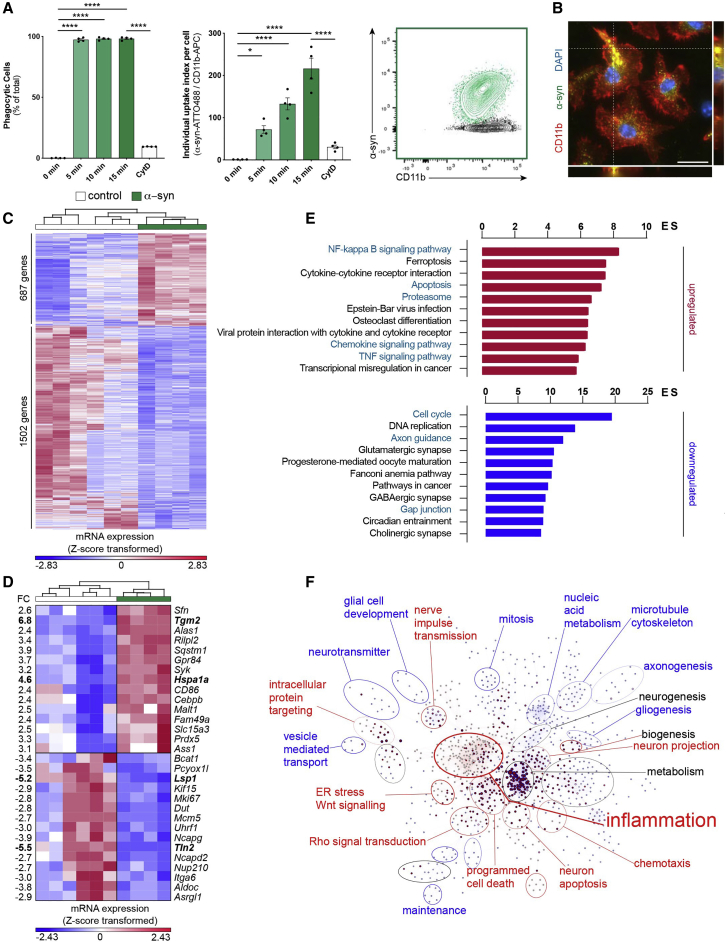
Uptake of α-syn fibrils results in the induction of an inflammatory profile (A) Quantification of the percentage of phagocytic cells (left) and the individual uptake index per cell (middle) after exposure to fluorescent α-syn fibrils (2 μM); n = 4. Diagram represents the α-syn uptake as measured by FACS (right). (B) Representative immunostaining showing the internalization of α-syn fibrils into CD11b^+^ microglia. (C) Heatmap of 2189 differentially expressed (DE) genes between control and α-syn-treated microglia. (D) Top 15 DE genes belonging to the α-syn signature identified by (Sarkar et al., 2020) in murine microglia plotted as Z-score transformed heatmap of gene expression values. (E) Bar chart of most enriched pathways for aggregated α-syn induced (red) and suppressed (blue) genes. (F) BiNGO enrichment map for DE genes between control and α-syn-treated microglia. Clusters were defined by the Cytoscape tool Wordcloud. FC, fold change; ES, enrichment score. Graphs represent the mean ± SEM and were analyzed by one-way ANOVA followed by Tukey’s multiple comparison post hoc test. ^∗∗∗∗^p < 0.0001, ^∗∗∗^p < 0.001, ^∗∗^p < 0.01, ^∗^p < 0.05. Scale bar: 20 μm. See also Figure S1; Table S1.

We first characterized α-syn uptake by exposing microglia for 5–15 min to fluorescent α-syn monomers (Figures S1F and S1G) or fibrils (Figures 1A and 1B) before uptake assessment by immunocytochemistry (ICC) and fluorescence-activated cell sorting (FACS) analysis. FACS analysis revealed a quick uptake of fibrillar α-syn with around 90% of cells being labeled after 5 min of exposure (Figure 1A). After 15 min 98% of cells contained α-syn fibrils with an increase of the individual α-syn content over time (Figure 1A). In contrast, α-syn monomers were engulfed to a much lower extent (Figure S1F). The uptake of α-syn monomers and fibrils was largely impeded by the phagocytosis inhibitor cytochalasin D (CytD) indicating active α-syn phagocytosis by microglia. The presence of α-syn aggregates inside the cytoplasm of microglia was confirmed using ICC (Figure 1B; Figure S1G).

To identify a specific α-syn-induced program and changes in microglia functions, transcriptome analysis of naive controls and cells that had been exposed to α-syn fibrils were performed. In total, 2,189 genes were differentially regulated (FC ± 1.5, false discovery rate [FDR] corrected p value 0.05) by α-syn in microglia (Figure 1C), of which 687 genes were induced and 1502 genes were suppressed. Making use of an already published dataset of α-syn treated microglia (Sarkar et al., 2020), we identified 170 α-syn signature genes in our dataset. Expression values of the top 15 induced or suppressed genes from the signature were plotted as a heatmap (Figure 1D). To further elaborate biological functions that are altered by α-syn, we performed pathway and gene ontology (GO) term analysis. We found that inflammation-related features were enriched, e. g. pathways for NFκB and TNFα, ferroptosis, apoptosis, and proteasome (Figure 1E). These results were further supported by the finding that 66%–71% of the differentially expressed (DE) genes belong to the interferome (Rusinova et al., 2013). Using the Biological Networks Gene Ontology tool (BiNGO) to create a network of induced and suppressed biological processes (Figure 1F), we confirmed that GO terms associated with inflammation and programmed cell death were highly enriched.

We confirmed the induction of an inflammatory profile of microglia exposed to α-syn fibrils previously (Scheiblich et al., 2021). Together, we have identified pathways that regulate pro-inflammatory and apoptotic mechanisms in microglia exposed to fibrillar α-syn.

### Intercellular transfer of fibrillar α-syn between microglia

**Figure 2:**
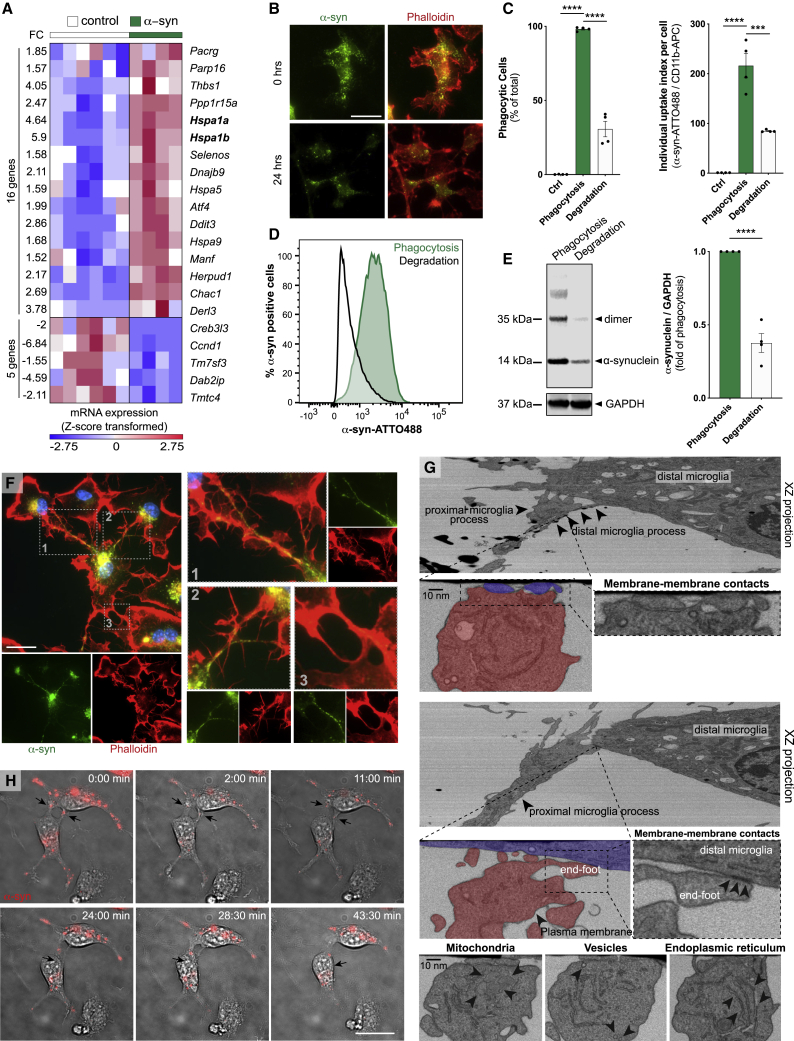
Microglia hesitate to degrade fibrillar α-syn and form a cellular network (A) Heatmap of Z-score transformed gene expression values for DE transcripts between α-syn treated microglia and controls related to the GO term “response to unfolded protein.” (B) Representative immunostaining of F-actin^+^ microglia before and after α-syn fibrils degradation. (C) Quantification of the number of cells (left) and the individual uptake index per cell (right) in microglia after α-syn phagocytosis (15 min) and degradation (24 h). n = 4. (D) Representative chart of fibrillar α-syn phagocytosis and degradation measured by FACS. (E) Immunoblot analysis and quantification of microglial lysates after fibrillar α-syn phagocytosis (15 min) and degradation (24 h). n = 4. (F) Representative immunostaining of microglia demonstrating various cellular F-actin^+^ connections containing α-syn. (G) Representative Electron Microscopy (FIB-SEM) images of membrane-to-membrane contacts of microglia. (H) Representative time-lapse recording demonstrating the transfer of α-syn aggregates from one microglia to another. Graphs represent the mean ± SEM and were analyzed by t test (E) or one-way ANOVA followed by Tukey’s multiple comparison post hoc test (C). ^∗∗∗∗^p < 0.0001, ^∗∗∗^p < 0.001, Scale bars: 20 μm. See also Figure S2.

GO term analysis (Figure 1F) emphasized that fibrillar α-syn regulates apoptotic processes and endoplasmic reticulum (ER) stress. Previous research determined a strong relationship between ER stress and protein degradation (Hetz and Papa, 2018). Interestingly, the majority of transcripts related to proteolysis and protein destabilization were not upregulated in response to α-syn. In contrast, most transcripts (65 out of 94) related to the biological function “response to unfolded protein” (GO term ID:0006986) were induced by α-syn of which 21 were differentially expressed (Figure 2A). Observing this upregulation, we wondered whether microglia would show any functional impairment of uptake or degradation. To assess α-syn processing, we allowed microglia to take up fibrils for 15 min and further incubated them for 24 h in α-syn-free medium. Using ICC (Figure 2B), FACS analysis (Figures 2C and 2D) and immunoblot analysis (Figure 2E) we found that about 40%–50% of α-syn remained undegraded after 24 h.

Using ICC, we found that microglia form a network of F-actin-positive membrane projections of various length and diameter that contain α-syn (Figure 2F). Electron microscopy imaging confirmed membrane-to-membrane contacts between neighboring microglia and the presence of different organelles inside the processes (Figure 2 G). Time-lapse analysis of microglia exposed to fluorescent α-syn showed that α-syn is transferred between microglia through two different types of intercellular α-syn transport mechanisms (Figure 2H; Figure S1H): One was characterized by relatively short and thick membrane projections (Figure 2 H; Video S1), which transferred large α-syn aggregates from one cell to another within 40–60 min. The second type was characterized by longer and thinner connections (Figure S1H; Video S2) that transferred small α-syn aggregates between microglia much faster in about 3 min. Importantly, α-syn was preferentially transferred from α-syn-loaded (donors) to α-syn-free (acceptors) cells (Figures S1I and S1J). Quantification of redistributed α-syn aggregates revealed a size preference of particles <1 μm (Figure S1K). Even though we found that untreated microglia formed some intercellular connections, α-syn induced the formation of those microglia-to-microglia connections, thereby increasing the number of microglial cell-to-cell contacts (Figure S1L).

Video S1. In vitro time-lapse video of microglia cells redistributing α-syn aggregates (black) from the upper cell to the lower cell. Detailed brightfield recordings can be found in Figure 2G, related to Figure 2G

Video S2. In vitro time-lapse video of microglia cells redistributing α-syn aggregates (black) from the upper cell to the lower cell. Detailed brightfield recordings can be found in Figure S1H, related to Figure S1H

**Figure 3:**
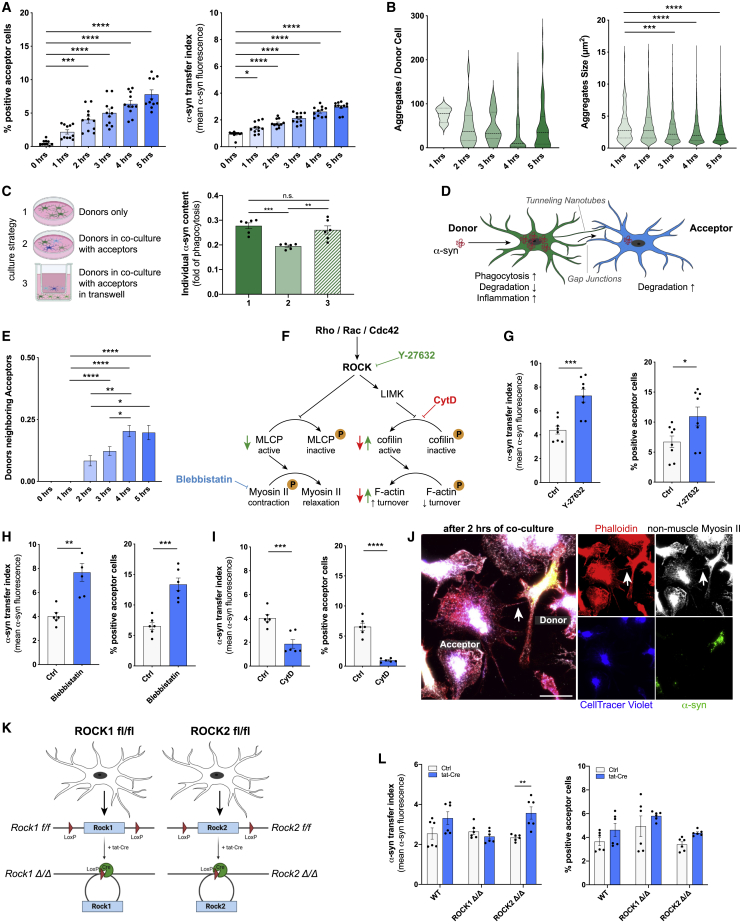
α-syn exchange between microglia is mediated by F-actin (A) Quantification of the number of α-syn-positive acceptors over time in co-culture with donors. n = 4. (B) Quantification of the number (left) and size (right) of α-syn aggregates in donors over time. n = 5. (C) Schematic drawing and quantification of α-syn degradation capacity of donors cultured alone (1), in co-culture with acceptors (2), or in co-culture with acceptors in a transwell (3). (D) Schematic depicting the α-syn transfer from donors to acceptors via tunneling nanotube-like structures and gap junctions. The drawing was created using BioRender.com and Adobe Illustrator. (E) Quantification of the number of donor-to-acceptor connections. n = 4 with 185–400 individual cells. (F) Schematic drawing of ROCK signaling and its downstream modulation of the F-actin cytoskeleton via LIM kinase (LIMK) and cofilin dephosphorylation, and the Myosin II actions via phosphorylation of the myosin light chain phosphatase (MLCP). The pharmacological inhibitors Y-27632, Blebbistatin, and Cytochalasin D (CytD) were used to block the downstream effects at different checkpoints. (G) Quantification of the effect of Y-27632 (10 μM) on α-syn transfer (left) and number of α-syn positive acceptors (right) at 5 h of co-culture. n = 4 with duplicate treatments. (H) Quantification of the effect of Blebbistatin (50 μM) on α-syn transfer (left) and number of α-syn positive acceptors (right) at 5 h of co-culture. n = 3 with duplicate treatments. (I) Quantification of the effect of CytD (5 μM) on α-syn transfer (left) and number of α-syn positive acceptors (right) at 5 h of co-culture. n = 3 with duplicate treatments. (J) Representative immunostaining revealing the presence of non-muscle Myosin II and F-actin inside cell-to-cell connections. (K) Schematic drawing of the generation of ROCK1- and ROCK2-knockout (Δ/Δ) microglia from ROCK1^flox/flox^ and ROCK2^flox/flox^ cells using a tat-Cre recombinase. (L) Quantification of the effect of ROCK1^Δ/Δ^ and ROCK2^Δ/Δ^ on α-syn transfer (left) and number of α-syn positive acceptors (right) at 5 h of co-culture. n = 3 with duplicate treatments. Graphs represent the mean ± SEM and were analyzed by one-way ANOVA followed by Kruskal-Wallis multiple comparison post hoc test for nonparametric data (A–E, L) or by a two-tailed t test (G–I). ^∗∗∗∗^p < 0.0001, ^∗∗∗^p < 0.001, ^∗∗^p < 0.01, ^∗^p < 0.05. Scale bars: 20 μm. See also Figures S2 and S3.

To further characterize this intercellular traffic, we set up cell culture experiments to determine if and how the observed α-syn transfer affects cellular functioning and survival. We differentially labeled donors containing fluorescent α-syn from acceptors that were loaded with a CellTracer and used co-cultures by which cells were either allowed to build direct cell-cell contacts or co-cultures separated by a porous membrane (pore size 3.0 μm). The proportion of acceptors capable of establishing direct contact with donors that became α-syn-positive was on average 8% after 5 h co-culture (Figure 3A). In contrast, we could not detect any α-syn-positive acceptors when cells were co-cultured without direct cellular contact (data not shown). Importantly, dose-response curve analysis indicated that only at higher concentrations (>0.25 μM) α-syn redistribution is a required step to reduce the individual cellular burden (Figure S2A). We adjusted the ratio of donor-to-acceptor cell numbers and analyzed changes in reactive oxygen species (ROS) levels. At a ratio of 1:3 (acceptors:donors), acceptors were not able to rescue donors anymore (Figure S2B). To prove that the observed α-syn within acceptors truly is a result of transfer, not of phagocytosis, we labeled donors with a CellTracer. Uptake quantifications of CellTracer-positive cellular debris excluded the ability that the observed α-syn within acceptors were a result of phagocytosis of donors (Figure S2C).

**Figure S4:**
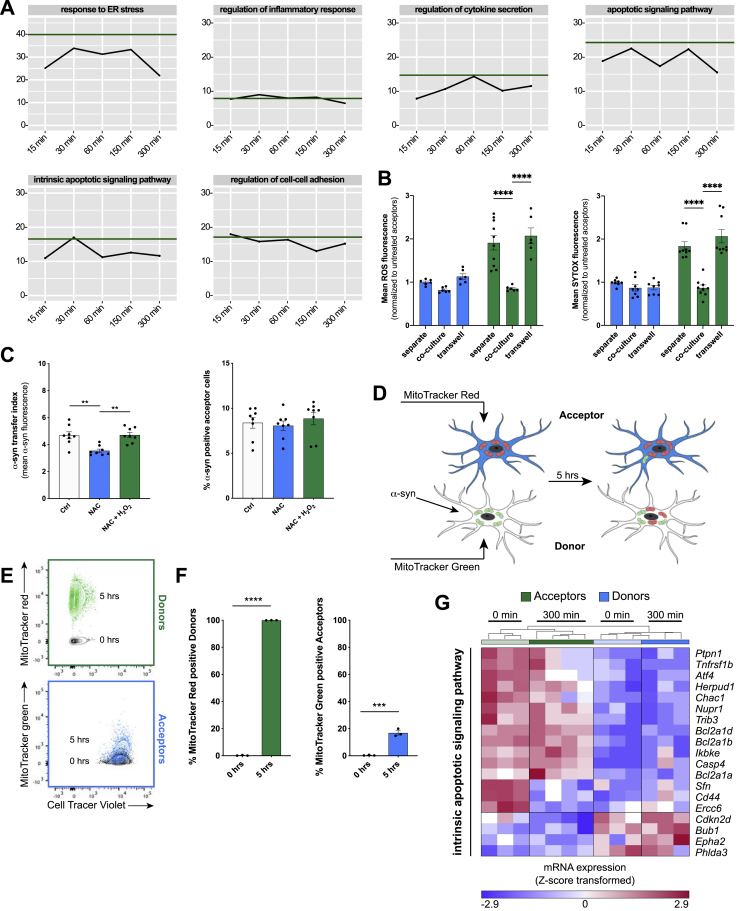
Transcriptomic analysis of microglia co-cultured in transwell inserts, related to Figure 4 (A) Enrichment Scores for selected GO terms in donors’ transcriptomes over the time of co-culture with acceptors using the transwell insert strategy to prevent direct cell-cell contact. Green line indicates the baseline ES at 0 min. (B) Quantification of the mean ROS fluorescence and percentage of ROS-positive cells of donors (green) and acceptors (blue) that were co-cultured for 5 h using the transwell insert strategy. n = 3 independent experiments. (C) Quantification of the α-syn transfer index from donors to acceptors (left) and the percentage of acceptors containing α-syn (right) after 5 h of co-culture upon treatment with the ROS scavenger N-Acetylcystein (NAC) and hydrogen peroxide (H_2_O_2_). n = 4 independent experiments with duplicated measurements. (D) Schematic illustrating the co-culture strategy used for experimental results presented in (E) and (F). The schematic was created using BioRender.com and Adobe Illustrator. (E and F) FACS analysis (E) and quantification (F) of the bidirectional transport of mitochondria from donors to acceptors and vice versa. n = 3 independent experiments. (G) Heatmap of Z-score transformed gene expression values for DE transcripts between α-syn treated microglia (“donors”) and control cells (“acceptors”) related to the GO term “intrinsic apoptotic signaling pathway“. All graphs are presented as mean ± SEM and were analyzed by two-way ANOVA (B) or one-way ANOVA followed by Tukey’s multiple comparison post hoc test (C, F). ^∗∗∗∗^p < 0.0001, ^∗∗∗^p < 0.001, ^∗∗^p < 0.01.

We used amyloid-β (Figure S2D) and tau (Figure S2E) aggregates as controls for other disease-associated proteins. Although we used a higher donor:acceptor ratio (1:1) in these experiments, we found that the uptake of both proteins led to a much lower protein exchange rate between microglia than α-syn (compare Figure 3A and Figures S2D and S2E).

To characterize the inter-microglial connections in more detail, we performed ICC against various docking proteins, cell-cell adherence proteins and cytoskeletal proteins. Interestingly, we observed an accumulation of Connexin 43 (Cx43) at the connection site (Figure S2F), a gap junction protein that has previously been described to connect interpericyte tunneling nanotubes (Alarcon-Martinez et al., 2020). Of note, α-syn aggregates trafficking between cells was detectable within these connections. Interestingly, not only the number and size of α-syn aggregates within the donors decreased with time (Figure 3B), but also the total α-syn burden was significantly reduced in donors co-cultured with acceptors (Figure 3C).

Together, these data indicate that co-culturing α-syn overloaded microglia with naive microglia results in the redistribution of α-syn aggregates to neighboring cells (Figure 3D).

### Intercellular α-syn fibrils transfer requires F-actin

Next, cytoskeleton dimensions within acceptors (Figures S2G–S2J) and donors (Figures S2K–S2N) were quantified over time. We found that the total cytoskeleton length of acceptors increased over time (Figure S2G), showing long and thin extensions into the nearby environment. While the total number of trunks slightly decreased (Figure S2H), the number of branches (Figure S2I) and the mean trunk to branch end distance (Figure S2J) largely increased. Similar cytoskeletal changes were observed in donors. However, since donors were layered on top of acceptors, these changes might arise from the attachment of the cells to the culture surface (Figures S2K–S2N). Moreover, the number of donor-to-acceptor connections significantly increased over time (Figure 3E).

Using the above described GO network, we identified Rho signal transduction as being upregulated in response to α-syn fibrils (Figure 1F). The Rho-kinase ROCK has been numerously identified as a key regulator of the cytoskeleton by downstream modulation of the actomyosin complex (Figure 3F). Using Y-27632 as a selective ROCK inhibitor, we found a markedly increased transfer of α-syn from overloaded donors to naive acceptors (Figure 3G). Treatment with the selective myosin II inhibitor Blebbistatin largely augmented α-syn transfer rate (Figure 3H), whereas inhibition of the F-actin turnover via CytD significantly impaired the transfer of α-syn (Figure 3I). In line with this, Y-27632 and Blebbistatin treatment induced the formation of a cellular network, whereas CytD largely inhibited this network formation (Figure S3A). Of note, this induction of α-syn exchange largely reduced the release of ROS from donors while CytD alleviates this effect (Figure S3B). SYTOX incorporation was not affected by these treatments (Figure S3C).

ICC confirmed the presence of the cytoskeletal marker’s myosin II and F-actin in these donor-acceptor connections (Figure 3J). Next, we used microglia derived from ROCK1^flox^ and ROCK2^flox^ mice that were treated with a tat-Cre recombinase to target the respective ROCK allele (Figure 3K). Interestingly, we found that ROCK1-knockout did not affect α-syn transfer, whereas ROCK2-knockout significantly increased the α-syn exchange rate (Figure 3L).

Together, these data indicate that activation of ROCK might inhibit the transfer of α-syn between microglia by downstream modulation of the actomyosin complex.

### Cell-to-cell transfer of fibrillar α-syn downregulates the inflammatory profile in microglia

**Figure 4:**
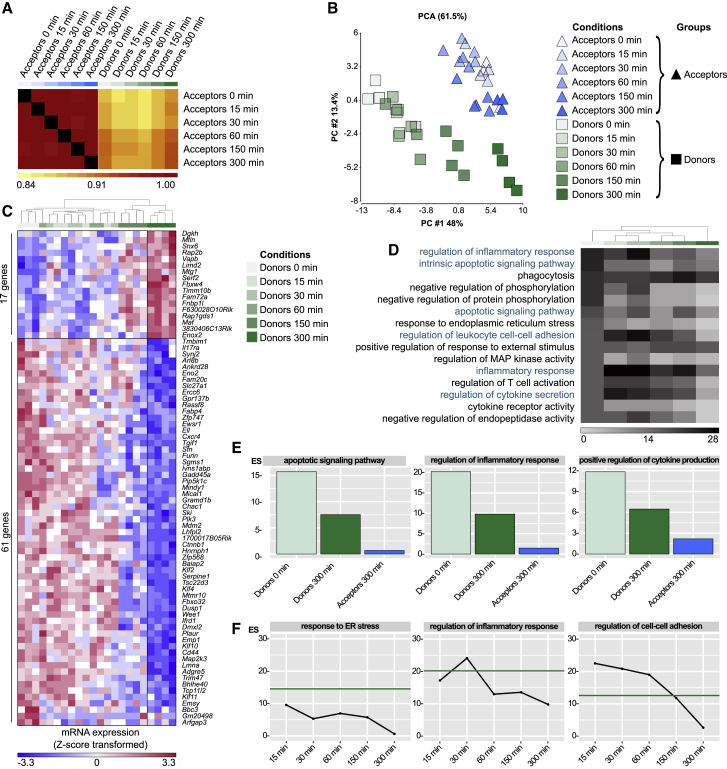
α-syn activated microglia are rescued from their inflammatory program by naive microglia (A) Heatmap of Pearson Correlation ρ value for the means of gene expression values in acceptors and donors over 5 h of co-culture. (B) 2D PCA and (C) heatmap of 78 DE genes in donors between 0 and 300 min of co-culture. (D) Heatmap for top 15 enriched GO terms sorted according to the Enrichment Scores (ES) in donors at 0 min up to 300 min of co-culture with acceptors. (E) Bar chart of ES for selected GO terms in donors and acceptors at 0 and 300 min. (F) ES for selected GO terms in donors’ transcriptomes over time in co-culture with acceptors. Green line indicates the baseline ES at 0 min. See also Figures S3 and S4.

To study the impact of the transfer of α-syn, we examined transcriptomic changes in donors and acceptors over time. Cells were co-cultured and sorted by FACS, and their RNA was collected and sequenced. A Pearson Coefficient ρ was calculated for each group and time point as quantitative measure for the similarity of the transcriptomes over the time of co-culture and visualized as heatmap (Figure 4A). While the transcriptomes of acceptors and donors showed high similarities within each group at all time points, the largest difference between acceptors and donors was observed before (0 min) co-culture. With increasing duration (5 h) of co-culture, donors were adopting an acceptor signature (Figure 4B).

DE gene analysis comparing transcriptomic changes in donors (0 versus 300 min) identified 17 upregulated and 61 downregulated genes (at least 1.5-fold change in either direction) (Figure 4 C; Table S1). To investigate biological functions altered by the transfer of α-syn, we performed two independent GO term enrichment analysis comparing mRNA profiles of acceptors and donors at 0 and 300 min of co-culture (Figures 4D and 4E). Before the co-culture, donors showed an enrichment of GO terms associated with “regulation of inflammatory response” (ES 20.2) and “apoptotic signaling pathway” (ES 15.7). This signature was ameliorated after 300 min of co-culture. It is worth noting that the transfer of α-syn to acceptors did not change their transcriptomic program. Time kinetic analysis revealed the downregulation of “regulation of inflammatory response” (Figure 4F). Interestingly, regulation of “cell-cell adhesion” (Figure S3D) was initially upregulated within the first hour of co-culture but declined over time (Figure 4F, right panel). Most importantly, these time kinetic changes (Figures S3E–S3G and Figure S4A) as well as the reduction in ROS release (Figure S4B) were absent when cell-to-cell contact between donors and acceptors was not permitted.

### Mitochondrial trafficking to escape cytotoxicity and cell death

**Figure 5:**
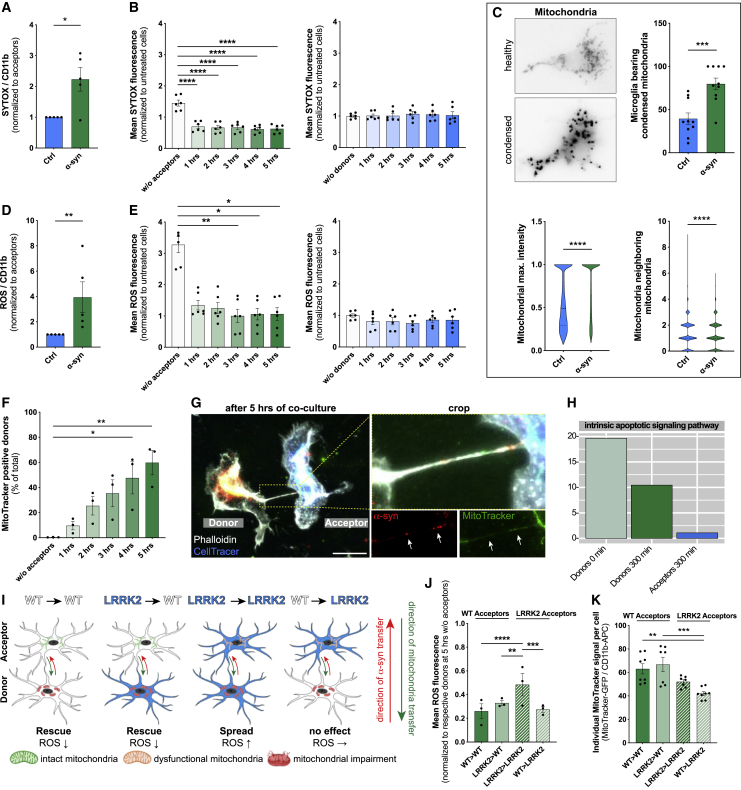
Mitochondrial propagation reduces microglial ROS (A) Quantification of the ratio of SYTOX penetration and intercalation into acceptors (Ctrl) and donors (α-syn). n = 5. (B) Quantification of the mean SYTOX penetration and intercalation into acceptors and donors over time in co-culture. n = 3 with dublicate measurements. (C) Representative immunostaining and quantification of healthy and condensed mitochondria. n = 5 with duplicate measurements. At least 20 individual cells were analyzed per n. (D) Quantification of the ratio of ROS production in naive acceptors (Ctrl) and α-syn-treated donors (α-syn). n = 5. (E) Quantification of the mean ROS production in acceptors and donors over time in co-culture. n = 3 with dublicate measurements. (F) Quantification of the exchange of mitochondria from acceptors to donors. n = 3. (G) Representative immunostaining demonstrating the presence of mitochondria (MitoTracker) and α-syn inside cell-to-cell connections. (H) Validation of enrichment analysis of the intrinsic apoptotic signaling pathway. (I) Schematic drawing of crossover co-culture experiments using WT and LRRK2 G2019S mutant microglia. The schematic was created using BioRender.com and Adobe Illustrator. (J) Quantification of the ROS production in crossover experiments using WT or LRRK2 mutant microglia. n = 3. (K) Quantification of the individual MitoTracker signal in crossover experiments with WT and LRRK2 G2019S microglia. Acceptors were stained for MitoTracker and their propagation toward donors was assessed. n = 3 with dublicate or triplicate measurements. Graphs represent the mean ± SEM and were analyzed by a two-tailed t test (A, C, D), or by one-way ANOVA followed by Tukey’s multiple comparison post hoc test (B, E, F, J) or by Dunn’s multiple comparison post hoc text (K). ^∗∗∗∗^p < 0.0001, ^∗∗∗^p < 0.001, ^∗∗^p < 0.01, ^∗^p < 0.05. Scale bar: 20 μm. See also Figures S4, S5, and S6.

Next, we studied the meaning of the described α-syn transfer on microglial function. Challenging microglia with α-syn compromised their plasma membrane, eventually leading to cell death, as suggested by the increased penetration of SYTOX into cells (Figure 5A). Interestingly, co-culturing donors with naive acceptors largely reduced the penetration of SYTOX into donors by about 50% (Figure 5B, left panel) without affecting the integrity of acceptors (Figure 5B, right panel). In parallel, we observed increased mitochondrial condensation (Figure 5C, upper panel and lower left panel) and disintegration of the mitochondrial network structure (Figure 5C, lower right panel), resulting in increased production of ROS (Figure 5D). Interestingly, co-culturing donors with naive acceptors largely reduced the production of ROS in donors (Figure 5E). To determine the function of ROS during α-syn redistribution, we used the ROS scavenger N-Acetylcystein (NAC) and H_2_O_2_ as a source of ROS (Figure S4C). Interception of ROS released by donors significantly reduced the amount of α-syn that underwent transfer. Remarkably, this effect was reversed by additional administration of H_2_O_2_ (Figure S4C), indicating that ROS might influence α-syn transfer.

**Figure S5:**
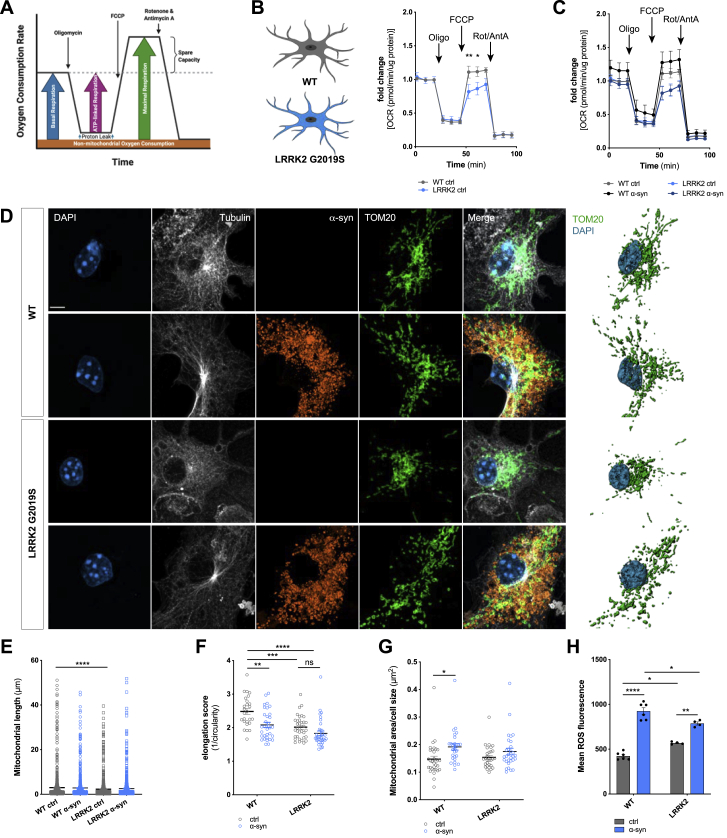
Effects of the LRRK2 G2019S mutation on mitochondrial fitness, related to Figure 5 (A) Schematic drawing of the Agilent Seahorse XF Cell Mito Stress Test profile, showing key parameters of mitochondrial function upon inhibition of the Electron Transport Chain complexes. (B) Oxygen Consumption Rate (OCR) of WT microglia (gray) and microglia carrying the LRRK2 G2019S mutation (blue) under basal conditions. n = 3 independent experiments. (C) Oxygen Consumption Rate (OCR) of WT microglia (gray/black) and microglia carrying the LRRK2 G2019S mutation (light blue/dark blue) upon treatment with 2 μM fibrillar α-syn for 24 h. n = 3 independent experiments. (D) Representative immunocytochemical staining and 3D reconstructions of WT microglia and microglia carrying the LRRK2 G2019S mutation demonstrating increased mitochondrial circulation (TOM20, green) following exposure to fibrillar α-syn (orange). (E–G) Quantification of the mitochondrial length (E), elongation score (F), and the mitochondrial area per cell size (G) of WT and LRRK2 G2019S mutant microglia. n = 3 independent experiments. (H) Quantification of the mean ROS release of WT microglia and microglia carrying the G2019S mutation under basal conditions and upon treatment with 2 μM α-syn fibrils for 24 h. n = 6 for WT and n = 4 for LRRK2 G2019S. All graphs are presented as mean ± SEM and were analyzed by two-way ANOVA. ^∗∗∗∗^p < 0.0001, ^∗∗∗^p < 0.001, ^∗∗^p < 0.01; ^∗^p < 0.05. Scale bar: 10 μm.

**Figure S6:**
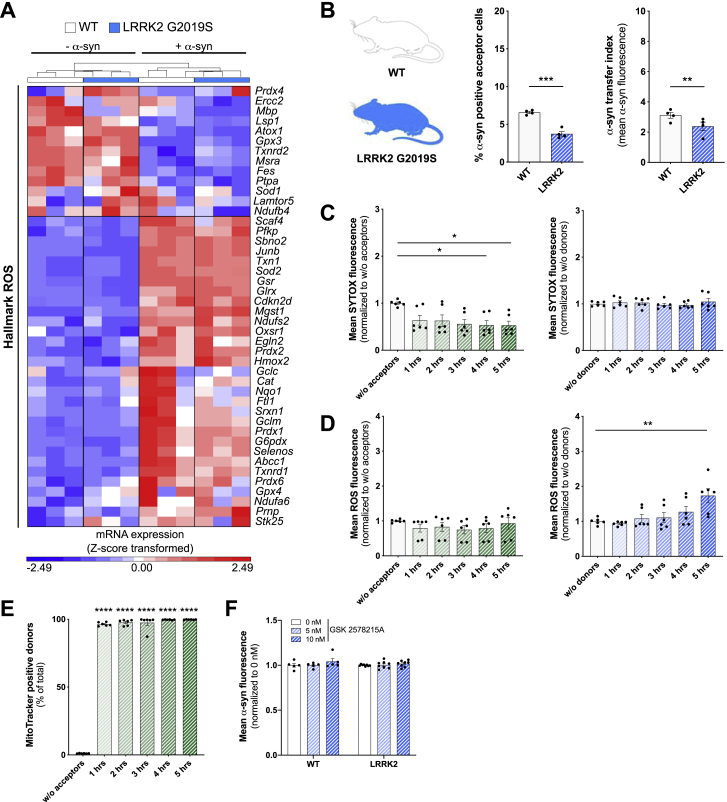
α-syn redistribution spread inflammation in microglia carrying the LRRK2 G2019S mutation, related to Figure 5 (A) Heatmap of Z-score transformed gene expression values for DE transcripts between WT and LRRK2 G2019S mutant microglia related to the Hallmark “ROS“. (B) Quantification of the α-syn transfer rate (left) and the number of α-syn positive cells (right) from donors to acceptors using WT (white) or LRRK2 G2019S microglia (blue). n = 4 independent experiments. (C) Quantification of the SYTOX penetration and intercalation into acceptors (blue) and donors (green) over time in co-culture using microglia carrying the LRRK2 G2019S mutation. n = 3 with duplicated treatments for all conditions. (D) Quantification of ROS production in acceptors (blue) and donors (green) over time in co-culture using microglia carrying the LRRK2 G2019S mutation. n = 3 with duplicated treatments for all conditions. (E) Quantification of the exchange of mitochondria from healthy acceptors to affected donors using microglia carrying the LRRK2 G2019S mutation. n = 3 independent experiments with triplicate treatments per condition. (F) Quantification of α-syn transfer from donors to acceptors using the LRRK2 inhibitor GSK 2578215A. n = 3 independent experiments with duplicated or triplicated measurements. Graphs are presented as mean ± SEM and were analyzed by t test (B) or one-way ANOVA followed by Tukey’s multiple comparison post hoc test (D right, E) or one-way ANOVA followed by a Dunn’s multiple comparison post hoc test (C right). ^∗∗∗∗^p < 0.0001, ^∗∗∗^p < 0.001, ^∗∗^p < 0.01, ^∗^p < 0.05.

In order to identify rescue strategies of microglia, we labeled mitochondria of naive acceptors with a MitoTracker and followed their fate over time (Figures 5F and 5G). Co-culturing donors and acceptors significantly increased the number of MitoTracker-positive donors (Figure 5F) up to 60% within 5 h. ICC revealed the presence of mitochondria and α-syn inside the cellular connections (Figure 5G). To prove a bidirectional intercellular mitochondrial exchange strategy, we differentially labeled mitochondria of acceptors red and those of donors green followed by a 5 h co-culture (Figure S4D). While all donors became positive for mitochondria donated from acceptors, around 20% of acceptors received mitochondria from donors (Figures S4E and S4F). Importantly, we found that the intrinsic apoptotic signaling pathway, that involves mitochondrial dysfunction, was downregulated in donors co-cultured for 5 h with acceptors (Figure 5H; Figure S4G). The most common genetic determinant causing PD has been identified to be the G2019S mutation in the *LRRK2* gene that has been associated with mitochondrial impairment. Using microglia from wild type (WT) or LRRK2 G2019S mutant mice, we checked for their oxygen consumption rate (OCR) (Figures S5A–S5C), mitochondrial morphology (Figures S5D–S5G), and ROS production (Figure S5H) in response to α-syn. As expected, LRRK2 microglia showed impaired mitochondrial fitness (Figure S5B) and a higher mitochondrial circulation rate (Figures S5D–S5G) than WT. Notably, α-syn increased the OCR (Figure S5C) and mitochondrial circulation (Figures S5D, S5F, and S5G) in WT but not LRRK2 microglia. Even though WT microglia had a lower basal ROS release than LRRK2 microglia, α-syn induced ROS in WT microglia to a greater extent than in LRRK2 cells (Figure S5H). Transcriptomic analysis revealed no changes in genes belonging to the hallmark “reactive oxygen species” expression between WT and LRRK2 microglia exposed to α-syn (Figure S6A).

LRRK2 microglia were significantly less efficient in transferring aggregated α-syn from affected donors to naive acceptors (Figure S6B). Similar to the above-mentioned (Figure 5B) experiments, we found that co-culturing LRRK2 donors with naive LRRK2 acceptors largely reduced the penetration of SYTOX into donors (Figure S6C). However, in contrast to WT microglia (Figure 5E), α-syn redistribution almost doubled the release of ROS by LRRK2 acceptors after 5 h of co-culture (Figure S6D). We found a strong exchange of mitochondria from naive acceptors to affected donors (Figure S6E). LRRK2 inhibition using GSK 2578215A had no effect on a-syn transfer in WT (Figure S6F).

We performed crossover experiments to prove whether WT microglia could rescue LRRK2 microglia by the redistribution of functionally intact mitochondria (Figure 5I). We co-cultured WT donors with LRRK2 acceptors and vice versa and measured ROS levels and the receipt of mitochondria (Figure 5I). Again, we found that co-culturing WT donors with WT acceptors (WT > WT) largely reduced their ROS production (Figure 5J). In contrast, LRRK2 acceptors were less efficient in rescuing LRRK2 donors (LRRK2 > LRRK2) with respect to their ROS levels (Figure 5J). However, most importantly, we found that co-culturing LRRK2 donors with WT acceptors (LRRK2 > WT) significantly reduced their ROS production (Figures 5I and 5J). In keeping with this, we found that LRRK2 and WT donors showed an impaired mitochondria exchange toward LRRK2 acceptors (LRRK2 > LRRK2 and WT > LRRK2), respectively, which was reversed when they were co-cultured with naive WT acceptors (LRRK2 > WT) (Figure 5K).

These data show that α-syn-loaded microglia transfer α-syn to naive microglia and, in parallel, receive functionally intact mitochondria from the acceptors thereby escaping from cytotoxicity and cell death. However, microglia carrying the LRRK2 G2019S mutation were not able to rescue neighboring cells, thereby inducing their own ROS level (Figure 5I;Figure S6D). These results indicate that dysregulated α-syn degradation in LRRK2 mutant microglia may represent one pathogenic factor by which mutations within LRRK2 cause familial PD.

### Evidence for exchange of α-syn in microglia from mice and human

**Figure 6:**
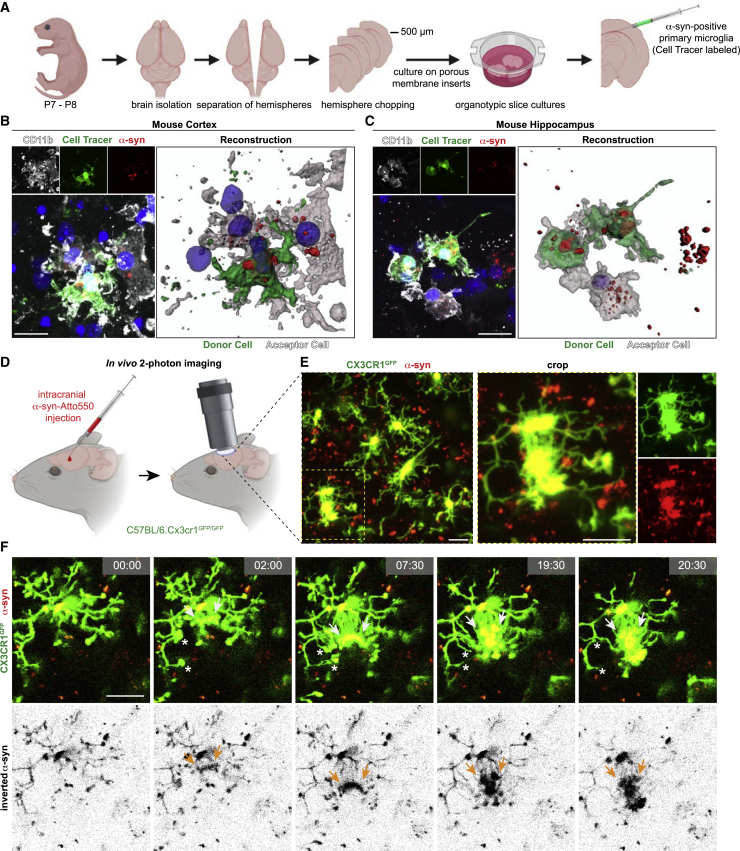
Cell-to-cell transfer of aggregated α-syn in microglia *in vivo* (A) Schematic illustrating the preparation of organotypic slice cultures (OSC) used for experiments shown in (B) and (C). (B and C) Representative immunostainings and 3D reconstructions of CellTracer labeled microglia containing α-syn injected into the cortex (B) or hippocampus (C) of an OSC connected to tissue-resident microglia with α-syn-positive inclusions. (D) Schematic illustrating *in vivo* 2-photon imaging used for experiments shown in (E) and (F). (E) Representative recording demonstrating the formation of a microglial network (Cx3cr1^GFP^) upon the injection of α-syn. (F) Representative time-lapse recordings demonstrating the transfer of α-syn between microglia via cellular membrane connections. Cellular connections were retracted (asterisk) once α-syn got transferred to the neighboring microglia. Schematics were created using BioRender.com. Scale bars: 20 μm. See also Figure S7.

**Figure S7:**
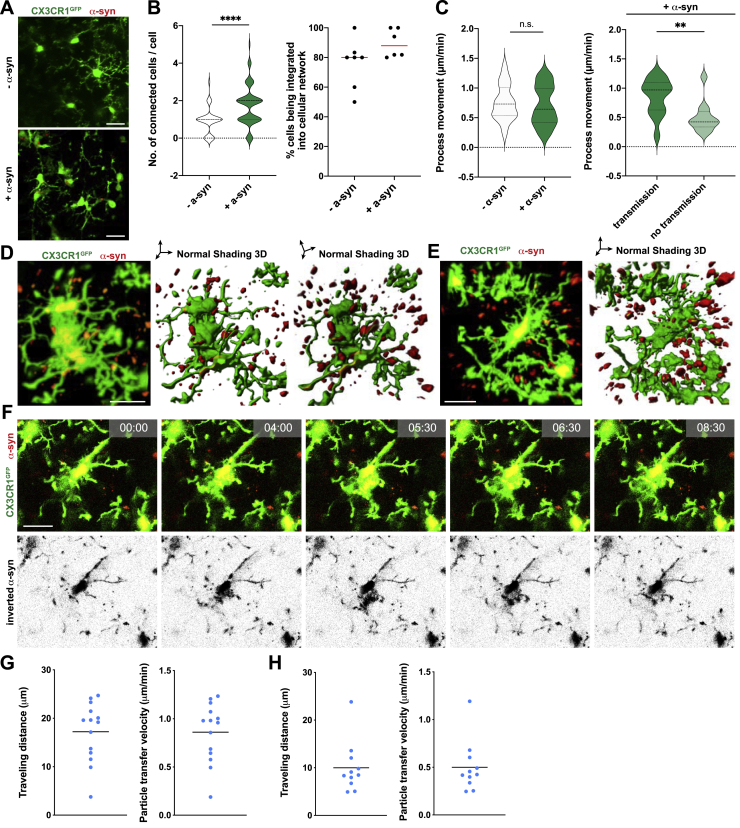
Formation of a functional microglial network, related to Figure 6 (A) Representative recording demonstrating the formation of a microglial network upon the intracranial injection of α-syn fibrils in Cx3cr1^GFP^ animals *in vivo*. (B) Quantification of the number of connected cells per individual cells and the percentage of microglia being integrated into a cellular network upon intracranial injection of α-syn fibrils in Cx3cr1^GFP^ animals *in vivo*. n = 2 animals per group with three to four randomly chosen areas that were analyzed for network formation. Interconnected microglia were counted manually. A total of at least 42 microglia were analyzed. (C) Quantification of process movement velocity of microglia recorded by 2-photon imaging with and without intracranial injection of α-syn fibrils in Cx3cr1^GFP^ animals (left panel). Quantification of process movement velocity of microglia not transmitting or transmitting α-syn aggregated to neighboring cells upon intracranial injection of α-syn fibrils. n = 25-30 individual processes were quantified. (D) Representative recording and 3D reconstruction of the cells in Figure 6F demonstrating the formation of a microglial network (Cx3cr1^GFP^, green) upon the injection of α-syn (red). (E) Representative recording and 3D reconstruction of distant microglia (Cx3cr1^GFP^, green) containing α-syn (red). (F) Representative time-lapse recording of microglia (Cx3cr1^GFP^, green) demonstrating that α-syn (red) is shuffled back into the cell soma when cells could not share the burden of α-syn by attaching to neighbor cells. (G) Quantification of particles that underwent transfer from one cell to another for their traveling distance and particle transfer velocity for cells shown in Figure 6F and Figure S14D. (H) Quantification of particles which transfer to a neighboring cell was unsuccessful for their traveling distance and particle transfer velocity for cells shown in Figure S14E and S14F. All graphs are presented as mean ± SEM and were analyzed by t test. ^∗∗∗∗^p < 0.0001, ^∗∗^p < 0.01. Scale bars: 20 μm.

To further elucidate the importance of the above-mentioned transfer mechanism, we set up a co-culture system of organotypic slice cultures (OSCs) in which we injected CellTracer-labeled and α-syn containing primary microglia (Figures 6A–6C). After 24 h of co-culture we observed various connections between the injected microglia and tissue-resident microglia. Interestingly, we found α-syn inside these connected resident microglia, suggesting a transfer of α-syn from the injected to the tissue-resident microglia (Figures 6B and 6C). In addition, we followed the fate of α-syn injected into the cortex of Cx3cr1^GFP+/−^ mice using *in vivo* 2-photon laser scanning microscopy (Figure 6D). Remarkably, we observed α-syn-positive microglia close to the injection site that formed a network containing various cell-to-cell connections (Figure 6E; Figures S7A, S7B, and S7D). Most importantly, we detected cells that extended their α-syn-containing processes to connect to neighboring cells (Figure 6F, arrows; Video S3), thereby increasing their process movement velocity (Figures S7C and S7G) compared to cells that do not transfer α-syn (Figures S7C and S7H). Following transmission of α-syn to their neighbors, the cell subsequently disconnected and retracted their processes (Figure 6F, asterisks). In addition to that, we observed cells that were unsuccessful in finding neighbors, shuffling α-syn back to their soma (Figures S7E and S7F; Video S4).

Video S3. In vivo 2-photon time-lapse video of CX3CR1-GFP microglia (green) transferring α-syn (red) to neighboring cells. Detailed descriptions of the transfer can be found in Figures 6E, 6F and S7D, related to Figures 6E, 6F, and S7D

Video S4. In vivo 2-photon time-lapse video of a CX3CR1-GFP microglia (green) not able to transfer α-syn (red) to a neighboring cell. Detailed descriptions of the transfer can be found in Figures S7E and S7F, related to Figures S7E and S7F

**Figure 7:**
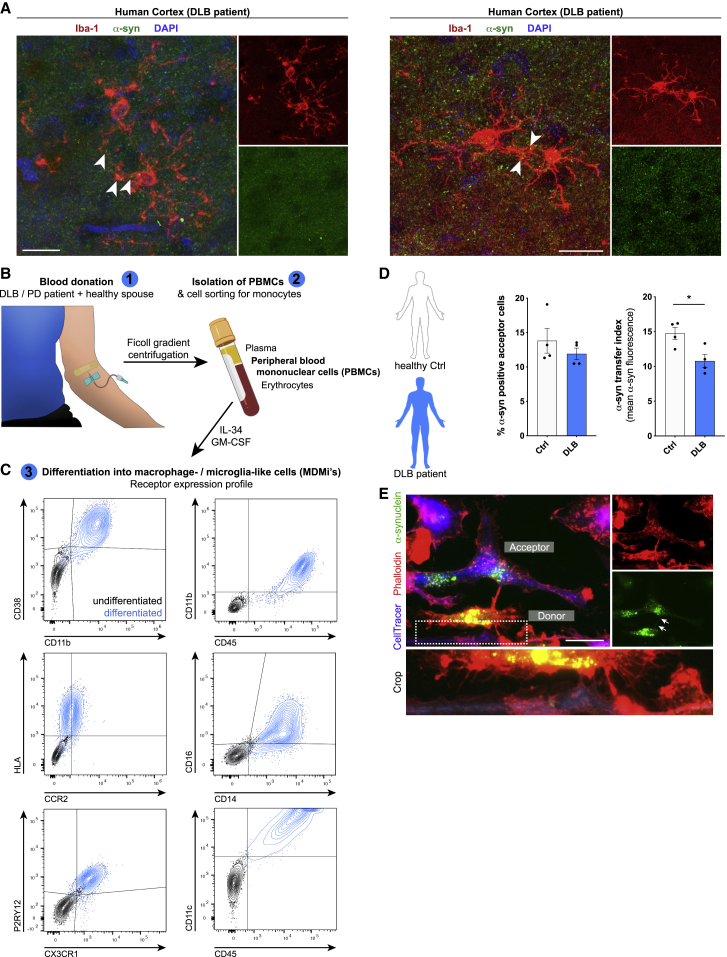
Cell-to-cell transfer of aggregated α-syn in human tissue and MDMis (A) Representative imaging of human cortical tissue from DLB patients. Samples were analyzed for Iba1 and α-syn. Arrowheads point toward α-syn aggregates containing microglia-to-microglia connections. (B) Schematic drawing of the isolation and differentiation of PBMCs into MDMis. The schematic was created using BioRender.com and Adobe Photoshop. (C) Charts representing the receptor expression profile of MDMis before and after differentiation as measured by FACS. (D) Quantification and comparison of the percentage and the transfer index of α-syn in human MDMis derived from healthy controls or DLB patients. n = 4. (E) Representative immunostaining of F-actin^+^ MDMis demonstrating the formation of membranous tubular connections between donors and acceptors. Graphs represent the mean ± SEM and were analyzed by t test. ^∗^p < 0.05. Scale bars: 20 μm. See also Figure S8.

**Figure S8:**
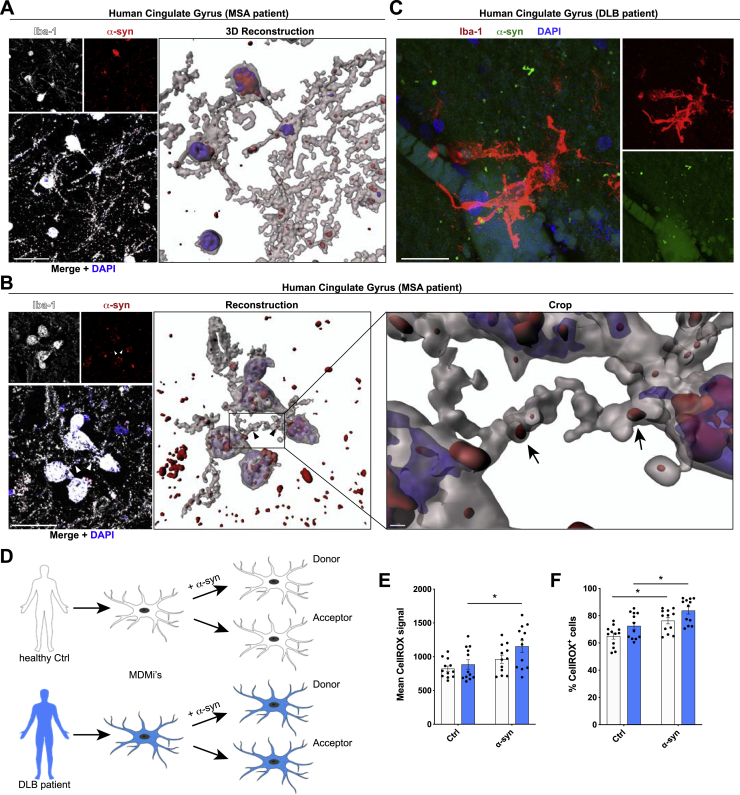
α-syn aggregates trigger a stronger ROS release in DLB patient-derived monocyte-derived microglia, related to Figure 7 (A) Representative immunohistochemical staining (left panel) and 3D reconstruction (right panel) of human cingulate gyrus samples from MSA patients. Samples were analyzed for Iba1-positive microglia (white) and α-syn (red). DAPI (blue) was used as nuclear counterstain. (B) Representative immunostaining (left panel) and 3D reconstruction (right panel) of human cingulate gyrus tissues from MSA patients. Samples were analyzed for Iba1-positive microglia (white) and α-syn (red). (C) Representative super-resolution imaging of human cortical tissues from DLB patients. Samples were analyzed for Iba1-positive microglia (red) and α-syn (red). (D) Schematic drawing of the use of patient monocytes-derived microglia. (E and F) Quantification of the mean CellROX signal (E) and the percentage of CellROX positive cells (F) using patient-derived monocyte-derived microglia treated for 24 h with 1 μM α-syn fibrils. All graphs are presented as mean ± SEM and were analyzed by a two-way ANOVA in conjunction with Sidak’s multiple comparison test. ^∗^p < 0.05. Scale bar: 20 μm.

Ultimately, using *post-mortem* human brain tissue samples of patients suffering from DLB (Figure 7A; Figure S8C) and multiple system atrophy (MSA) (Figures S8A and S8B), we found several microglia filled with aggregated α-syn. Interestingly, many of these cells were connected by α-syn-containing cell-to-cell connections. This led us to speculate that the exchange of α-syn between microglia might also occur in human patients. We used macrophage-/microglia-like cells that were differentiated from peripheral blood mononuclear cells (PBMCs) isolated from DLB patients or their healthy spouses (Figure 7B). Successful differentiation of the isolated PBMCs was confirmed by checking the expression pattern of various macrophage/microglia markers (Figure 7C). Remarkably, co-culturing donors and naive acceptors (Figure S8D) resulted in a significantly attenuated transfer rate of α-syn in DLB patient-derived cells compared to their healthy counterparts from control individuals (Figure 7D). ICC confirmed the presence of tubular, α-syn-containing cell-to-cell connections between donors and acceptors (Figure 7E). Exposure of patient-derived MDMi’s to α-syn significantly elevated the ROS production (Figures S8E and S8F) compared to baseline (untreated) levels, thus indicating that ROS might influence the transfer of α-syn between cells.

Together, our data present evidence that microglia have the ability to actively connect to neighboring cells to share the amount of cytotoxic protein accumulations e.g., as a strategy to provide aid and support of protein degradation and to attenuate inflammatory reactions. This mechanism has been proven by us to be conserved over different *in vitro*, *ex vivo*, and *in vivo* model systems.

## Discussion

Aggregated α-syn accumulations and microglial activation represent key pathological hallmarks of synucleinopathies. Even though α-syn aggregates are first found in neurons, evidence suggests that spreading of pathology and neuronal cell death will expose these proteins to surrounding microglia. Microglia are responsible for the clearance of misfolded and aggregated proteins from the brain and represent the main drivers of inflammatory processes within the CNS, and their activation has been widely observed in PD (for review see Lecours et al., 2018). Once activated, microglia initiate a range of inflammatory responses and phagocytic clearance mechanisms of the respective protein aggregates. Since inflammatory events within the brain may affect cellular functions of surrounding cells, this may cause quantitative and qualitative differences of the microglial phagocytic clearance of protein aggregates.

### Membranous tubular connections transfer fibrillar α-synuclein between microglia

Effective microglial clearance of misfolded and aggregated proteins may play an important role in neurodegenerative diseases. Aggregated α-syn has been shown to be released from neurons and is detectable in biological fluids including plasma and CSF (El-Agnaf et al., 2003; Mollenhauer et al., 2011). Microglia are the main cell type responsible for the clearance of aggregated proteins within the CNS, thereby limiting the spreading of pathology (George et al., 2019). We therefore assessed the efficacy of microglial clearance of α-syn fibrils. Microglia quickly took up the α-syn fibrils in a time-dependent manner (Figures 1A and 1B) resulting in the initiation of inflammatory reactions and apoptosis (Figures 1C–1F; Scheiblich et al., 2021). However, microglia seemed to hesitate to degrade fibrillar α-syn (Figures 2A–2E). As a consequence, accumulation of α-syn aggregates within the cells was observed. Several studies provide evidence for impaired clearance mechanisms in PD leading to the idea that pathogenic α-syn can block its own clearance, resulting in protein deposition (Cuervo et al., 2004; Snyder et al., 2003). Alternatively, the microglial activation state may alter its intracellular capacity to effectively degrade internalized protein aggregates (Lee et al., 2008). Defects in degradation may force microglia to initiate alternative aggregate removal routes such as autophagy (Choi et al., 2020) or transfer of α-syn to neighboring cells to share the burden of protein degradation.

Within the CNS there is an extensive cellular cross-talk ongoing that mediates intercellular communication over long range distance in order to protect the brain from damage. Cell-to-cell communication plays an important role in various physiological and pathological conditions. Cells can communicate in a variety of ways, ranging from soluble factor secretion to direct cell-to-cell contacts. Recent observations have demonstrated that immune cells including macrophages, monocytes, and natural killer cells can be connected through cellular processes, which allows for the exchange of cytoplasmic molecules (Panasiuk et al., 2018).

Here, we demonstrate that α-syn fibrils can traffic between microglia via direct cell-cell contact (Figure 2G), resulting in improved clearance (Figures 2H, 3B, and 3C; Figures S1 and S2). Intercellular α-syn transmission has been observed between neurons, astrocytes, and pericytes but has not yet been reported for microglia (Abounit et al., 2016; Dieriks et al., 2017; Emmanouilidou et al., 2010; Freundt et al., 2012; Masuda-Suzukake et al., 2013; Rostami et al., 2017). Currently, two connection systems that allow direct exchange of cytosolic factors between connected cells have been described: gap junctions and tunneling nanotubes. According to their definition (Okafo et al., 2017; Onfelt et al., 2006) we suggest that microglia use both systems for intercellular α-syn aggregate exchange (Figure 3D). Interestingly, untreated microglia displayed several intercellular connections, which were further increased in number after the administration of α-syn (Figure S1L). Thus, this additional and *de novo* formation of intercellular microglial connections was induced by the presence of α-syn. *In vitro*, microglial donors efficiently transfer α-syn aggregates to neighboring acceptors, thereby requiring direct cellular contact (Figure 2; Figure S1). Our data are in line with a previous study, demonstrating that microglial communication through functional gap junctions, induced by inflammatory cytokines, plays a key role in the elaboration of the inflammatory response (Eugenín et al., 2001).

### ROCKing α-syn clearance mechanisms in microglia

α-syn induced cytoskeletal changes (Figures S2G–S2N) and the formation of a microglial network (Figure S1L) resulted in an efficient reduction of the total α-syn burden in donors (Figures 3B and 3C). These cytoskeletal changes are most likely mediated by cytoskeletal forces that are provided by the polymerization of globular actin into filaments and microtubules, both of which are involved in the formation of TNTs (Sisakhtnezhad and Khosravi, 2015). Filamentous actin-containing membrane protrusions have been recently shown to play an essential role in long-range intercellular communication (Bukoreshtliev et al., 2009; Dilsizoglu Senol et al., 2019), and its actions in cell-cell α-syn and mitochondria transmission via TNTs has been described previously (Rostami et al., 2017). The Rho-kinase ROCK has been repeatedly identified as a key regulator of the cytoskeleton by downstream modulation of the actomyosin complex (Amano et al., 2010; Maekawa et al., 1999), and its activation in microglia likely represent a contributing pathogenic factor in PD (Barcia et al., 2012; Gentry et al., 2016; Saal et al., 2017; Tönges et al., 2012, 2014). In line with this, ROCK inhibition completely prevented microglial activation and fully restored dopaminergic neuronal cell number in the MPTP mouse model of PD (Barcia et al., 2012) likely by attenuating α-syn aggregation (Tatenhorst et al., 2016). The involvement of the Rho-kinase ROCK pathway in TNT formation and organelle transport has been described elsewhere (Arkwright et al., 2010; Dupont et al., 2018; Keller et al., 2017). Using Y-27632 as a selective inhibitor of ROCK, we found a markedly increased transfer of aggregated α-syn between cells (Figure 3G), indicating the involvement of downstream targets of ROCK in the transfer mentioned above. In addition, inhibition of myosin II largely increased the transfer of fibrillar α-syn between cells (Figure 3H), whereas exposure to the potent inhibitor of actin polymerization CytD largely inhibited aggregated α-syn exchange (Figure 3I) indicating that myosin II inhibits the required actions of F-actin. Using ROCK1^flox^ and ROCK2^flox^ microglia, we found that ROCK2 knockout significantly increased the exchange of α-syn between microglia (Figures 3K and 3L). Interestingly, ROCK1 has been described to mainly act on the actomyosin complex, whereas ROCK2 has been shown to modulate actin polymerization (Shi et al., 2013).

Thus, ROCK2 appears to orchestrate protein aggregate degradation in microglia, thereby contributing to the formation of an “on-demand” microglial degradation network.

### Mitochondria donation attenuate microglial inflammatory reactions

A role for mitochondrial dysfunction in the pathogenesis of PD has long been appreciated as several PD-associated genes have been linked to mitochondrial pathways. In keeping with this, genetic studies provided further support for the involvement of mitochondrial impairment in PD and identified 14 mitochondrial function-associated genes that increase the risk of developing PD (Billingsley et al., 2019). Dysfunctional mitochondria undergo fragmentation resulting in the enhanced release of ROS, thereby further amplifying mitochondrial alteration. Here, we found that α-syn fibrils induced the production of ROS (Figure 5D), resulting in a compromised plasma membrane (Figures 5A and 5B) and mitochondrial network disintegration (Figure 5C) likely leading to microglial apoptosis and cell death. Most importantly, co-culturing donors with naive acceptors allowed sharing the burden of aggregated α-syn, largely reduced the α-syn-induced ROS production, and rescued cells from cell death (Figures 4 and 5E) by shuffling intact mitochondria from naive to affected microglia (Figures 5F and 5G), thereby attenuating the intrinsic apoptotic signaling pathway (Figure 5H). Horizontal transfer of mitochondria via TNTs, microtubule-based transport strategies, and vesicles (Rodriguez et al., 2018) has recently been shown between different cell types, with incorporation of the donated mitochondria into the mitochondrial network of recipient cells (Cho et al., 2012; Hayakawa et al., 2016; Islam et al., 2012; Spees et al., 2006). Importantly, most of the work on mitochondrial transfer deals with the rescue of damaged cells (Ahmad et al., 2014; Cho et al., 2012; Islam et al., 2012) as it seems to be the case in our study. We detected mitochondria from naive acceptors in the aforementioned cellular connections as well as in the cytoplasm of the donors (Figure 5G), indicating that the donation of mitochondria is linked to improved microglial survival and the attenuation of the microglial inflammatory profile (Figure 4). Around 20% of acceptors received mitochondria from donors (Figures S4E and S4F). Whether and how this bidirectional mitochondria transfer might be part of a rescue strategy, e.g., via transmitophagy (Davis et al., 2014), needs to be further elucidated.

The most common genetic cause of PD is the G2019S mutation in *LRRK2* that upregulates LRRK2 kinase activity. Just recently, the LRRK2 G2019S mutation has been shown to inhibit the degradation of α-syn in an *in vitro* model of PD (Hu et al., 2018) and disrupts mitochondrial depletion via mitophagy (Hsieh et al., 2016). Moreover, LRRK2 has been biologically linked to pathways regulating inflammation and phagocytosis (Wallings and Tansey, 2019), highlighting a critical role of LRRK2 in inflammation. We found that microglia from WT mice were more efficient in redistributing fibrillar α-syn between cells than microglia from mice harboring the LRRK2 G2019S mutation (Figure S6B). This suggests that mutant *LRRK2* might impact the formation of a functional microglial degradation network, resulting in cytotoxic α-syn accumulation. However, further studies are required to understand in detail the role of *LRRK2* in the establishment of membranous networks between microglia upon exposure to fibrillar α-syn. In addition, exchange of α-syn increased ROS levels in LRRK2 acceptor cells (Figure S6D, right panel). Most importantly, co-culturing WT acceptors with fibrillar α-syn-loaded LRRK2 donors reduced their inflammatory ROS levels (Figures 5I and 5J) by the donation of functionally intact mitochondria (Figures 5I and 5K). Together, our data support the idea that microglia can establish an “on-demand” functional network for efficient clearance of ingested pathological aggregates in order to attenuate and control microglial inflammatory reactions. LRRK2 G2019S might impact the formation of a functional rescue network thereby propagating inflammation in PD.

### Bridging microglia to improve fibrillar α-syn clearance

Using *ex vivo* OSC experiments, we demonstrated that primary microglia establish connections with tissue-resident microglia and efficiently transfer fibrillar α-syn to the latter (Figures 6A–6C). In addition to microglia, we found α-syn accumulations in cells negative for CD11b (Figure 6C, right side nucleus). Just recently, it has been described that microglia and astrocytes may interact in α-syn clearance, thereby exchanging α-syn among each other (Rostami et al., 2021). Thus, it seems possible that microglia may transfer α-syn to other cell types, e.g., astrocytes, in our OSC experiments. Moreover, *in vivo* 2-photon imaging revealed the formation of a functional network with various microglia being connected to each other and α-syn fibrils that were redistributed among cells through cellular connections (Figures 6D and 6E; Video S3). Thus, our study provides evidence for the formation of a functional microglial network to efficiently share the burden of pathogenic α-syn *in vivo*. By analyzing brain sections from patients suffering from DLB or MSA, we detected cellular connections between α-syn-bearing microglia (Figure 7A; Figures S8A–S8C), further supporting our idea of an active transfer mechanism of pathogenic α-syn between cells within the human brain.

To mimic what may occur in the human brain, we differentiated MDMi's (Figures 7B and 7C) from a cohort of DLB-diseased volunteers and their healthy spouses and assessed the potential of microglial cells to share the burden of pathogenic α-syn (Figures 7D and 7E). We successfully proved the ability of human MDMi's to share aggregated α-syn with neighboring cells through membranous tubular connections (Figures 7D and 7E). Most importantly, the potential of sharing aggregated α-syn with neighboring cells lacking those aggregates was attenuated in cells derived from DLB-patients. In these cells, α-syn aggregates enhanced the production of ROS (Figures S8E and S8F) indicating that ROS might, at least to some extent, negatively influence the intercellular transfer of α-syn aggregates between cells. However, whether and how ROS might modulate the transfer of aggregated α-syn from one cell to another requires further investigations.

### Limitations of the study

In conclusion, our observations suggest that microglia can share aggregated α-syn with neighboring cells devoid of such pathogenic aggregates, thereby lowering the individual burden of degradation. This may represent a process by which microglia support each other, as in a community, to quickly and efficiently degrade misfolded and aggregated proteins. Most importantly, lowering the burden of α-syn aggregates in affected cells reduced the production of ROS and its cytotoxic effects by the donation of mitochondria from naive cells. However, this study has potential limitations that need to be addressed in future research. Indeed, we did not analyze in detail the cellular mechanisms involved in the formation of the membranous structures allowing α-syn and mitochondria transfer, including contact formation and fusion of membranes. Furthermore, we have not investigated the intracellular degradation pathway of α-syn in microglia and its modulation upon receiving support from neighboring cells. Although we were able to show the exchange of α-syn between cells *in vivo*, we could not prove that this transfer occurred via TNTs. Basically, the question arises whether TNTs can be found in a similar manner *in vivo*, then in the cell culture dish. Since TNTs are very fine and thin cell-cell connections, we do not know whether they could be dissolved by our 2-photon laser microscopy *in vivo* techniques. To our knowledge, there is currently only one publication describing the presence of TNTs between cells of the CNS (Alarcon-Martinez et al., 2020). Also, we were not able to record any transfer from heavily burden cells to naive cells, which was due to the fact that the α-syn was already spreading during the preparation of the cranial window. Thus, further studies are required to determine and understand the detailed mechanisms of cell connections and the molecular mechanisms responsible for pathogenic α-syn assemblies transport between neighboring microglia.

Importantly, while our study describes microglia-to-microglia TNT connections which are important to distribute the burden of α-syn degradation, future studies will have to investigate whether similar contacts and mechanisms exist between microglia and neurons.

## Star★Methods

### Key resources table

**Table:** 

REAGENT or RESOURCE	SOURCE	IDENTIFIER
**Antibodies**		

7-AAD	BD Biosciences	Cat# 559925
Alexa Fluor™ 647 Phalloidin	Invitrogen	Cat# A22287
alpha Tubulin Monoclonal Antibody (DM1A)	Thermo Fisher Scientific	Cat# 62204; RRID: AB_1965960
Anti Iba1, Rabbit antibody	FUJIFILM Wako Pure Chemical Corporation	Cat# 019-19741; RRID:AB_839504
Anti-non-muscle Myosin IIB antibod antibody	abcam	Cat# ab204358; RRID: AB_2737410
APC anti-human CD11c antibody	BioLegend	Cat# 337208; RRID:AB_1279066
APC anti-mouse/human CD11b	Bio Legend	Cat# 101212; RRID: AB_312795
APC mouse anti-CD36 antibody	BD Biosciences	Cat# 550956; RRID:AB_398480
Beta Actin antibody	Proteintech	Cat# 20536-1-AP; RRID:AB_10700003
BV421 mouse anti-human CD16 antibody	BD Biosciences	Cat# 562874; RRID:AB_2716865
BV605 anti-human CD192 (CCR2) antibody	BioLegend	Cat# 357214; RRID:AB_2563876
BV605 mouse anti-human CD11b antibody	BD Biosciences	Cat# 562721; RRID:AB_2737745
Connexin 43 Antibody	Cell Signaling	Cat# 3512; RRID: AB_2294590
FITC anti-human CX3CR1 antibody	BioLegend	Cat# 341606; RRID:AB_1626272
goat anti-mouse-AlexaFluor594	Invitrogen	Cat# A11020; RRID: AB_141974
goat anti-rat-AlexaFluor488	Invitrogen	Cat# A11006; RRID: AB_141373
HLA-DR, DP, DQ antibody	BD Biosciences	Cat# 555558; RRID:AB_395940
IRDye® 680LT Donkey anti-Mouse IgG (H + L)	LI-COR Biotechnology	Cat# 926-68022; RRID: AB_10715072
IRDye® 800CW Goat anti-Rabbit IgG (H + L)	LI-COR Biotechnology	Cat# 926-32211; RRID: AB_621843
N-cadherin Polyclonal Antibody	Thermo Fisher Scientific	Cat# PA5-19486; RRID: AB_10979609
PE anti-human P2RY12 antibody	BioLegend	Cat# 392104; RRID:AB_2716007
PE anti-mouse/human CD11b antibody	Bio Legend	Cat# 101207; RRID: AB_312790
PE-Cy7 mouse anti-CD14 antibody	BD Biosciences	Cat# 557742; RRID:AB_396848
Polyclonal Rabbit anti-Human TNFAIP2 Antibody	LSBio	Cat# LS-C386457
Purified (azide-free) anti-alpha-Synuclein, 103-108 antibody	BioLegend	Cat# 807801; RRID: AB_2564730
Rabbit Anti-beta Catenin Monoclonal Antibody, Unconjugated, Clone E247	Abcam	Cat# ab32572; RRID: AB_725966
Rabbit Anti-GAPDH Antibody	Sigma-Aldrich	Cat# G9545; RRID:AB_796208
Rat anti mouse CD11b antibody	Serotec by Bio-Rad	Cat# MCA711; RRID: AB_321292
Recombinant Anti-RAB8A antibody [EPR14873]	Abcam	Cat# ab188574; RRID: AB_2814989
Rock-2 (30-J) antibody	Santa Cruz Biotechnology	Cat# sc-100425; RRID:AB_1129154
Texas Red™-X Phalloidin	Thermo Fisher Scientific	Cat#T7471

**Biological Samples**		

Human *post-mortem* brain sections	Tübingen Brain Bank	N/A
Human *post-mortem* brain sections	DZNE Brain Bank Bonn	N/A

**Chemicals, Peptides, and Recombinant Proteins**		

4’,6-Diamidino-2’-phenylindol-dihydrochloride (DAPI)	Thermo Fisher Scientific	Cat# 62247
ATTO-488 NHS-ester	Atto-Tec GmbH	Cat# AD 488-35
ATTO-550 NHS-ester	Atto-Tec GmbH	Cat# AD 550-35
Buprenorphine hydrochloride	Indivior Eu Ltd.	PZN# 345928
Calcium chloride	Sigma-Aldrich	Cat# 499609
Cefotaxime	MIP pharma	PZN# 3916283
CellTracer™ Violet	Thermo Fisher Scientific	Cat# C34557
Dexamethasone	Jenapharm	PZN# 8704321
Dulbecco‘s Modified Eagle‘s Medium	GIBCO by Thermo Fisher Scientific	Cat# 31966047
Dulbecco‘s Phosphate-Buffered Saline	GIBCO by Thermo Fisher Scientific	Cat# 14190169
Fetal Bovine Serum	LIFE Technologies	Cat# 10270106
Glucose	Sigma-Aldrich	Cat# G7528
GlutaMAX	GIBCO	Cat# 35050061
Halt Protease Phosphatase Inhibitor Cocktail	Thermo Fisher Scientific	Cat# 78441
Ketamine	Ratiopharm	PZN# 7538837
Lipopolysaccharide from *Escherichia coli* K12	InvivoGen	Cat# tlrl-eklps
N2-Supplement	GIBCO by Thermo Fisher Scientific	Cat# 17502048
Normal goat serum	Abcam	Cat# ab7481
Normal Horse Serum	Abcam	Cat# ab139501
NuPAGE® 4–12% Bis-Tris gel	Invitrogen	Cat# NP0323BOX
Paraformaldehyde	Sigma-Aldrich	Cat# P6148
Penicillin/Streptomycin	GIBCO by Thermo Fisher Scientific	Cat# 15070063
Phosphate-Buffered Saline	Biochrom GmbH	Cat# L 182-10
Poly-D-lysine hydrobromide	Sigma-Aldrich	Cat# P6407
Poly-L-lysine hydrobromide	Sigma-Aldrich	Cat# P1524
Proteinase K	Thermo Fisher Scientific	Cat# 10181030
Sodium bicarbonate	Sigma-Aldrich	Cat# S5761
Sodium dodecyl sulfate (SDS)	Carl Roth	Cat# CN30.2
Superfrost ultra plus slides	Thermo Fisher Scientific	Cat# J3800AMNZ
Thioflavin T	Sigma-Aldrich	Cat# T3516-25G
Tissue-Tek® Optimal Cutting Temperature (OCT) compound	Sakura by Thermo Fisher Scientific	Cat# 4583
Trypsin-EDTA (0.5%), no phenol red	LIFE Technologies	Cat# 15400054
Xylazine	Serumwerk Bernburg	PZN# 10124950

**Critical Commercial Assays**		

Mouse IL-1 beta/IL-1F2 DuoSet ELISA	R&D Systems	Cat# DY401
Mouse IL-10 DuoSet ELISA	R&D Systems	Cat# DY417
Mouse IL-6 DuoSet ELISA	R&D Systems	Cat# DY406
Mouse TNF-alpha DuoSet ELISA	R&D Systems	Cat# DY410
Pierce™ BCA Protein Assay kit	Thermo Fischer Scientific	Cat# 23225
Proteome Profiler Mouse Cytokine Array Kit, Panel A	R&D Systems	Cat# ARY006
RNeasy Micro Kit	QIAGEN	Cat# 74004
Pierce LAL Chromogenic Endotoxin Quantification Kit	Fisher Scientific	Cat# 88282
XF Cell Mito Stress Test	Agilent	Cat# 103015-100

**Deposited Data**		

GEO Dataset	https://www.ncbi.nlm.nih.gov/geo/	GEO accession number: GSE152100
GEO Dataset	https://www.ncbi.nlm.nih.gov/geo/	GEO accession number: GSE166127

**Experimental Models**		

E.coli BL21 DE3 CodonPlus cells	Aligent Technologies	Cat# 230245
Human: peripheral blood mononuclear cells (PBMCs)	University of Bonn - Medical Center	N/A
Mouse: C57BL/6	Charles River Laboratories	RRID: IMSR_JAX:000664
Mouse: C57BL/6 Cx3cr1^GFP^	The Jackson Laboratory	RRID: IMSR_JAX:005582
Mouse: C57BL/6 LRRK2 G2019S	Taconic Biosciences	RRID: IMSR_TAC:13940
Mouse: C57BL/6 ROCK1^flox^	kind gift of Prof. Henneberger	N/A
Mouse: C57BL/6 ROCK2^flox^	kind gift of Prof. Henneberger	N/A

**Software and Algorithms**		

CellProfiler	Broad Institute of Harvard and MIT	v3.1.8
FACSDIVA™ software	Becton Dickinson	N/A
Fiji ImageJ	Wayne Rusband	v2.0.0-rc-69/1.52n
FlowJo	FlowJo, LLC	v3.05470
ggplot2	CRAN	v3.2.1
Graph Pad Prism	GraphPad Software Inc.	v7.0e and v8.0
Image Studio, v5.2	LI-COR Biosciences	N/A
Imaris	Bitplane by Oxford Instruments plc	v9.2.1
NIS-elements	Nikon	AR 4.20.03
Partek Genomics Suite and R	Parket Inc.	v3.5.0
tidyr	CRAN	v1.0.2

**Other**		

BD FACSCANTOII	BD Biosciences	equipment
HiSeq2500	Illumina	equipment
Infinite M200 Pro	TECAN	equipment
Leica TCS SP8 STED	Leica	equipment
Nikon Eclipse Ti fluorescence microscope	Nikon	equipment
ODYSSEY CLx Imaging System	LI-COR Biotechnology	equipment
Schick driller C1 device	Schick GmbH	equipment
Ti:Sapphire 2-photon laser scanning microscope	Nikon	equipment
XFe-24 Extracellular Flux Analyzer	Agilent	equipment
Zeiss Laser Scan Microscope 800	Carl Zeiss	equipment

### Resource availability

#### Lead contact

Further information and requests for resources and reagents should be directed to and will be fulfilled by the Lead Contact, Michael T. Heneka (michael.heneka@ukbonn.de).

#### Materials availability

This study did not generate new unique reagents.

### Experimental model and subject details

#### Animals

Wild-type (WT, Charles River Laboratories, Inc., Wilmington, MA, USA), LRRK2 G2019S (Taconic Biosciences, New York, USA), Cx3cr1^GFP^ (The Jackson Laboratory, Bar Harbor, ME, USA), Rock1^flox^ and Rock2^flox^ (both kindly provided by Prof. Henneberger) animals were all of the C57BL/g genetic background. Mice were housed under standard conditions at 22°C and a 12 h light-dark cycle with free access to food and water. Animal care and handling was performed according to the guidelines of animal welfare as laid down by the German Research Council (DFG) and approved by the local ethical committees.

#### Primary microglia generation

Primary microglia cells were isolated by the method of (Giulian and Baker, 1986). Briefly, brains from neonatal mice (P0-P3; mixed gender) were stripped of the meninges and dissociated using mechanical shearing and trypsin (Life Technologies, Carlsbad, CA, USA). Cells of two brains were plated on poly-L-lysine (PLL, Sigma-Aldrich by Merck KGaA, Darmstadt, Germany) coated T75 culture flasks (Greiner bio-one, Kremsmünster, Austria) and cultivated in DMEM (GIBCO by Thermo Fisher Scientific, Waltham, MA, USA) supplemented with 10% heat-inactivated fetal calf serum (FCS; GIBCO) and 1% penicillin/streptomycin (P/S; GIBCO). On the next day, cells were washed three times with DPBS (GIBCO) to remove cellular debris and cultured with DMEM supplemented with 10% FCS, 1% P/S and 1% L929 conditioned medium as a source of growth factors. After 7-10 days loosely attached mature microglia were shaken off the astrocytic layer with a repetition of the harvesting procedure all two to three days for up to three times. For experiments, primary microglia were seeded into well plates and allowed to adhere overnight in DMEM complemented with 1% N-2 supplement (GIBCO) before experiments were performed.

#### Differentiation of patient-derived cells

The use of patient-derived peripheral blood mononuclear cells and its differentiation into monocyte-derived microglia/macrophages has been approved by the ethics committee of the University Hospital Bonn – Medical Center.

MDMi’s were prepared based on previous protocol (Sellgren et al., 2017) with some modifications. As we did not find any changes between male and female MDMi’s, subjected pools of patients from synucleinopathies (male n = 3, age 65-80; female n = 2, age 69-76) and control patients (male n = 1, age 75; female n = 3, age 78-81) were analyzed.

For peripheral blood mononuclear cells (PBMCs) isolation patient and control subject blood samples, collected in EDTA, were diluted in PBS (equivalent blood volume) and transferred on top of the Ficoll layer (GE Healthcare Cat#17-5442-02), 1/3rd volume of diluted blood. The tubes were then centrifuged at 400*xg*, at RT, acceleration (slow) and brake (slow) for 30min. After centrifugation, the upper layer was discarded and the PBMCs layer at the interphase was collected in a fresh 50ml Falcon tube. The cells were washed twice with PBS and counted. Monocytes were then isolated using the Pan Monocyte Isolation Kit (Miltenyi Biotec Cat#130-096-537) according to manufacturer instructions. For MDMi generation, monocytes were seeded on Matrigel (Corning Cat#356231) coated 6 wells plates, 1 × 10^6^/well in differentiation medium (RPMI 1% P/S, 10% FBS, IL34 100 ng/mL (R&D systems Cat#5265-IL-010/CF); GM-CSF 10 ng/mL (R&D systems Cat#215-GM-050/CF)), 2ml/well. Medium were added or half-replaced every other day. On day 14, cells were collected and plated in RPMI + 1% P/S + 10% FBS. The next day, medium was replaced with RPMI + 1%P/S. Cells were ready for experiment on day 17. Further experiments were performed in RPMI + 1%P/S.

#### Organotypic Slice Culture (OSC)

Brains from postnatal day 7 wild-type mice (mixed gender) were dissected and cultured by the method of (Croft and Noble, 2018). Briefly, animals were rapidly sacrificed using large scissors and heads were transferred to ice-cold slice culture dissection buffer for brain isolation (1.25 mM KH_2_PO_4_ pH 7.4, 124 mM NaCl, 3 mM KCl, 8.19 mM MgSO_4_, 2.65 mM CaCl_2_, 3.5 mM NaHCO_3_, 10 mM glucose, 2 mM ascorbic acid, 39.4 μM ATP in ultrapure H_2_O, sterile filtered (0.2 μm)). Brains were transferred onto fresh filter paper, placed on the cutting stage of a McIlwain tissue chopper (Campden Instruments Ltd., Loughborough, UK) and sliced into 500 μm thick sections. Brain slices were then transferred to Organotypic cell culture inserts (Millicell® provided by Merck) and cultured in 1 mL slice culture medium (36.7% Basal medium eagle (BME, Thermo Fisher Scientific), 36.7% Neurobasal-A Medium (Thermo Fisher Scientific), 1% GlutaMAX (GIBCO by Thermo Fisher Scientifics), 0.033% insulin (Life Technologies), 0.5% P/S, 25% heat inactivated horse serum (Abcam)). 24 h after the slicing culture medium was replaced by fresh medium followed by medium changes every two days. After 7 days in culture, 0.5 μl primary microglia containing fluorescent α-syn fibrils were injected to the cortex or the hippocampus region. OSCs were fixed 24 h after injection using 4% PFA for 1 h and stained for microglia as described below.

#### Human tissue samples

The use of human *post-mortem* brain sections provided by the Tübingen Brain Bank and the DZNE Brain Bank Bonn has been approved by the ethics committee of the University Hospital Bonn – Medical Center.

Please see the “Immunohistochemistry” section for further information on sample processing of the brain sections.

### Method details

#### α-synuclein assembly generation

Human wild-type α-syn was expressed in *E. coli* BL21 DE3 CodonPlus cells (Agilent Technologies, Santa Clara, CA, USA) and purified as described previously (Ghee et al., 2005). To assemble human wild-type α-syn into the fibrillar polymorph “Fibrils,” the protein (100 μM) was incubated in 50 mM Tris–HCl, pH 7.5, 150 mM KCl at 37°C under continuous shaking in an Eppendorf Thermomixer set at 600 r.p.m for 5 days (Bousset et al., 2013). The assembly reaction was followed by withdrawing aliquots (20 μl) from the assembly reaction at different time intervals, mixing them with Thioflavin T (10 μM final) and recording the fluorescence increase on a Cary Eclipse Fluorescence Spectrophotometer (Varian Medical Systems Inc., Palo Alto, CA, USA) using an excitation wavelength = 440 nm, an emission wavelength = 480 nm and excitation and emission slits set at 5 and 10 nm, respectively. Following assembly reaction, fibrils were fragmented to an average length of 42-52 nm. The molecular mass of fragmented fibrils was then determined by analytical ultracentrifugation. Fibrils were made on average of ∼8300 monomers which means that a working concentration of 2 μM equivalent monomeric α-syn corresponds to a particles (fibrils) concentration of 0.24 nM (2000/8300 = 0.24). All α-syn preparations were quantified for endotoxin levels as described previously (Grozdanov et al., 2019; Peelaerts et al., 2015) to prove that endotoxin levels were below 0.02 endotoxin units/μg (EU/μg) using the Pierce LAL Chromogenic Endotoxin Quantification Kit.

To label α-syn fibrils with extrinsic fluorophores, the fibrils were centrifuged twice at 15,000 *g* for 10 min and re-suspended twice in PBS at 1,446 g/L and two molar equivalents of ATTO-488 NHS-ester or ATTO-550 NHS-ester (Atto-Tec GmbH, Siegen, Germany, #AD 488-35 and #AD 550-35, respectively) fluorophore in DMSO were added. The mix was incubated for 1 h at room temperature. The labeling reactions were arrested by addition of 1mM Tris pH 7.5. The unreacted fluorophore was removed by a final cycle of two centrifugations at 15,000 *g* for 10 min and resuspensions of the pellets in PBS. The fibrillar nature of α-syn was assessed by Transmission Electron Microscopy (TEM) after adsorption of the fibrils onto carbon-coated 200 mesh grids and negative staining with 1% uranyl acetate using a Jeol 1400 transmission electron microscope. The images were recorded with a Gatan Orius CCD camera (Gatan, Pleasanton, CA, USA). The resulting α-syn fibrils were fragmented by sonication for 20 min in 2 mL Eppendorf tubes in a Vial Tweeter powered by an ultrasonic processor UIS250v (250 W, 2.4 kHz; Hielscher Ultrasonic, Teltow, Germany) to generate fibrillar particles with an average size 42-52 nm as assessed by TEM analysis.

#### Phagocytosis assay

To assess microglial phagocytosis, primary microglia (3.5 × 10^5^ cells/well) were seeded to 24-well plates and allowed to adhere overnight. Microglia were treated with 1 μM Atto488-labeled α-syn fibrils and incubated for 5-15 min. Phagocytosis was stopped by one washing steps with PBS to remove free α-syn and cells were harvested using 0.5% trypsin (GIBCO). Cells were then labeled with the APC anti-mouse/human CD11b antibody (1:100; #101212, BioLegend, San Diego, CA, USA) for 30 min in FACS solution (PBS supplemented with 2% FCS) on ice. Following labeling, cells were collected, resuspended in 300 μl ice cold FACS solution, and measured by flow cytometry using the FACS CANTO II and the FACSDIVA software (Becton Dickinson, Heidelberg, Germany). Phagocytosis was then analyzed and quantified using FlowJo, LLC (v3.05470, Ashland, OR, USA).

#### Cell-to-cell transfer of α-syn aggregates

To determine the transfer of α-syn aggregates from one cell to another, we used differentially labeled donor and acceptor cells. Acceptor cells were seed at a density of 300,000 cells per well into a 24-well plate and allowed to adhere overnight. Donor cells were seed at a density of 2 Mio. cells per well in a 6-well plate. On the next day, acceptor cells were washed once with PBS followed by the labeling with CellTracer Violet (Thermo Fisher Scientific) according to the manufacturer’s protocol. Labeling reaction was stopped with medium containing 10% serum, discarded and replaced by serum free medium. In parallel, donor cells were incubated for 30 min with 1 μM ATTO488-labeled α-syn fibrils in serum free medium followed by 2 washing steps in PBS before getting trypsinized using 0.5% trypsin (GIBCO). Donor cells were collected and pelleted for 5 min at 300xg. Donor cells were resuspended in fresh serum free medium and added to the acceptor cells in a 1:3 ratio (donors:acceptors) at different time points (1-5 h). For experiments on patient-derived cells, MDMi’s of each patient were divided into donor and acceptor subgroups and co-cultured as described above. Importantly, acceptor cells of one patient were co-cultures with the respective donor cells of the same patient (Figure S8 D). Cell-to-cell transfer of α-syn aggregates was stopped by one washing steps with PBS and cells were harvested using 0.5% trypsin. Blocking solution containing PBS and FCS (1:1 ratio) was applied for 10 min on ice. Cells were then labeled with the APC anti-mouse/human CD11b antibody (1:100) for 30 min in FACS solution on ice. Following labeling, cells were collected, resuspended in 200 μl ice cold FACS solution, and measured by flow cytometry using the FACS CANTO II and the FACS DIVA software (Becton Dickinson, Heidelberg, Germany). Cell-to-cell transfer of α-syn aggregates was then analyzed and quantified using FlowJo, LLC (v3.05470, Ashland, OR, USA).

#### Flow cytometric analysis of monocytes-derived microglia (MDMi)

After collection of MDMi on day 14, cells were washed in PBS + 2% BSA. Cells were incubated in PBS + 2% BSA for 30 min, then with fluorochrome-conjugated antibodies (5-20μl/10^6^ cells) for 30 min in the dark on ice: CD11b-BV605 (BD Biosciences Cat#562721), CD11c-APC (BioLegend Cat#337208), CD14-PE-Cy7 (BD PharMingen Cat#557742), CD16-BV421 (BD Biosciences Cat#562874), CD36-APC (BD PharMingen Cat#550956), CD45-APC-Cy7 (BD Biosciences Cat#557833), CCR2-BV605 (BioLegend Cat#357214), CX3CR1-FITC (BioLegend Cat#341606), HLA-DP, DQ, DR-FITC (BD PharMingen Cat#555558), P2RY12-PE (BioLegend Cat#392104). After staining, cells were washed in PBS + 2% BSA and incubated with 7AAD (BD Biosciences Cat#559925) for 10 min. Cells were then washed, resuspended in 100 μl of PBS + 2% BSA and processed with the BD FACS CANTO II. Data were analyzed using FlowJo.

#### Measurement of cytokine secretion

Cytokine release was determined using the mouse IL-1 beta/IL-1F2 DuoSet ELISA (DY401, R&D Systems, Minneapolis, MN, USA), mouse TNF-alpha DuoSet ELISA (DY410, R&D Systems), mouse IL-6 DuoSet ELISA (DY406, R&D Systems), mouse IL-10 DuoSet ELISA (DY417, R&D Systems), and mouse CXCL2/MIP-2 DuoSet ELISA (DY452, R&D Systems). Primary microglia (7.5 × 10^4^ cells/well) were seed into 96-well plates and allowed to adhere overnight. Microglia were primed for 3 h prior to experiments with 10 ng/mL lipopolysaccharide (LPS; InvivoGen, San Diego, CA, USA) before cells were washed with DPBS and treated with 2 μM α-syn fibrils. Supernatants were assayed after 24 h treatment according to the manufacturer’s protocol. Optical density was determined at 450 nm photometrically with a microplate reader (Infinite M200, Tecan, Männedorf, Switzerland). Concentrations of the secretion of the different cytokines were calculated by interpolation using a respective cytokine specific standard curve.

#### Oxygen consumption rate measurements

Cellular oxygen consumption rate (OCR) was measured using an XFe-24 Extracellular Flux Analyzer together with the XF Cell Mito Stress Test (all Seahorse Agilent). Cells were seeded at a density of 150,000 cells/well 48 h before the measurement. On the day of the experiment, cells were first switched to Seahorse XF base medium containing 1 mM pyruvate, 10 mM galactose as well as 2 mM glutamine, and then equilibrated for 60 min in a CO_2_-free incubator at 37°C. Following three OCR measurements at baseline, the ATP synthase inhibitor oligomycin (0.5 μM), the mitochondrial uncouppler FCCP (2 μM) and the complex I/III inhibitors rotenone/antimycin A (0.5 μM) were sequentially added. Once bioenergetic recordings were completed, cells were collected and lysed in RIPA buffer (Sigma Aldrich), supplemented with protease and phosphatase inhibitors (Roche), and protein concentrations were determined via Bradford assay. OCR values were then normalized to the respective protein contents.

#### Measurement of ROS production

The generation of reactive oxygen species (ROS) was determined using the CellROX® Deep Red Flow Cytometry Assay Kit (LIFE Technologies) according to the manufacturer’s protocol. In brief, donor cells from cell-to-cell transfer experiments were treated with Atto550-labeled α-syn fibrils and incubated as described above. After microglia were labeled with the PE anti-mouse/human CD11b antibody (1:100, Bio Legend) for 15 min in FACS solution on ice, cells were collected and cultured in medium containing 500 nM of the CellROX® Deep Red reagent for 30 min at 37°C. During the final 15 min of staining 1 μM SYTOX® Blue Dead Cell stain solution was added to the cells. After that cells were directly assessed by flow cytometry.

#### RNA sequencing

To determine transcriptomic changes caused by the transfer of α-syn aggregates from one cell to another, we used the above described co-culture and labeling strategy (see “cell-to-cell transfer of α-syn aggregates” section). Cell were collected before and 15, 30, 60, 150, and 300 min after co-culture of donor and acceptor cells. Co-cultures were sorted back into their original “donor” and “acceptor” cell populations by flow cytometrical cell sorting and RNA was collected using 700 μl Trizol.

For the isolation of RNA up to 500,000 cells per sample were lyzed in Trizol. Isolation of bulk RNA was performed with the RNeasy Micro Kit (QIAGEN). Library production for 3′-mRNA sequencing was performed with up to 125 ng purified RNA according to the manufacturers’ protocol and sequenced on a HiSeq2500 (Illumina) with a sequencing depth of 15 Mio reads per sample (NGS Core Facility, University Hospital, Bonn, Germany). Reads were aligned with STAR (v2.5.3a) against the murine reference genome mm10. Transcripts were quantified with the Partek E/M algorithm and further processed for normalization in R with the DEseq2 algorithm. Batch effects derived by independent experiments were removed in the Partek Genomics Suite (v7.18.0402). The dataset was further optimized by flooring transcripts with minimal gene counts at least to £1 and the exclusion of transcripts with a mean expression £10 in every test condition. Differentially expressed genes were determined for α-syn versus untreated control microglia by a two-way-ANOVA including the experiment as batch effect (fold-change |1.5|, FDR-adjusted p value £0.05). Data visualization and biological interpretation were performed with the Partek Genomics Suite and R (v3.5.0) packages ggplot2 (v3.2.1) for graphical visualization of expression data and tidyr (v1.0.2) for data wrangling.

#### Western blot

For lysate collection, primary microglia (2 × 10^6^) were cultured in 6-well plates under control conditions or in medium containing 2 μM α-syn fibrils for 24 h before cells were washed and scraped off the well with ice cold PBS containing 1x protease and phosphatase inhibitor cocktails (Thermo Fisher Scientific). After pelleting the cells for 5 min at 10,000 g PBS was completely removed and cells were lysed in ice cold RIPA buffer (50 mM Tris-HCl, 1% Triton X-100, 0.5% Na deoxycholate, 0.1% sodium dodecyl sulfate (SDS), 150 mM NaCl, pH 8.0) containing 1x protease inhibitor cocktails for 15 min on ice. Lysates were centrifuged 5 min at 4°C and 10,000 g and supernatants were frozen and kept in −20°C until use.

Cell lysates were separated by a NuPAGE® 4%–12% Bis-Tris Gel (Invitrogen by Thermo Fisher Scientific) and transferred to a nitrocellulose blotting membrane (0.2 μm; GE Healthcare Life Sciences, Freiburg, Germany). Membranes were washed with Tris-buffered saline supplemented with Tween-20 (TBST, 10 mM Tris-HCl, 150 mM NaCl, 0.05% Tween-20, pH 8.0). Membrane surface was blocked with 3% BSA in TBST for 30 min at RT. Membranes were then incubated with the mouse anti-α-synuclein antibody (1:1,000; BioLegend) and rabbit anti-GAPDH (1:1,000; Sigma-Aldrich) overnight at 4°C. After three washing steps á 5 min with TBST the fluorescent near-infrared secondary antibodies IRDye® 800CW Goat anti-Rabbit IgG (H + L) (1:10,000 in 3% BSA, LI-COR Biosciences, Lincoln, NE, USA) and IRDye® 680LT Donkey anti-Mouse IgG (H + L) (1:10,000 in 3% BSA, LI-COR Biosciences) were applied for 30 min at RT. Proteins were then visualized with the Odyssey CLx Imaging System (LI-COR Biosciences) and quantified using Image Studio (LI-COR Biosciences).

#### Immunocytochemistry

Cultures were fixed in 4% paraformaldehyde (PFA, Sigma-Aldrich) dissolved in PBS (Biochrom GmbH, Berlin, Germany) for 15 min and permeabilized by washing them three times for 5 min with PBS containing 0.1% Triton X-100 (PTX). Blocking solution containing PTX and 5% normal goat serum (Vector Laboratories, Burlingame, CA, USA) was applied for 30 min. The primary antibodies mouse anti-α-synuclein (1:500; BioLegend), rat anti-CD11b (1:250; Serotec by Bio-Rad), or mouse anti-non-muscle Myosin IIB (1:500; abcam) were applied for 1 h followed by three washing steps. The secondary antibodies goat anti-mouse-AlexaFluor594 (1:250; Invitrogen) and goat anti-rat-AlexaFluor594 (1:250; Invitrogen) were applied for 30 min. Texas Red-X Phalloidin (1:1,000, Thermo Fisher Scientifics) was applied for 20 min without prior blocking steps. 4’,6-Diamidino-2’-phenylindol-dihydrochloride (DAPI, Sigma-Aldrich) was used for nuclear counterstaining at 0.1 mg/mL for 20 min in PBS. Images were taken using a 60x oil-objective.

#### Imaging and analysis of mitochondrial morphology

Fluorescently-stained cells were imaged using a confocal microscope with Airyscan (Zeiss LSM800) and a 63x oil immersion objective. Z stacks at a thickness of 0.5 μm between each focal plane were taken from at least 20 cells per condition. Maximum intensity projections of original images were generated in ImageJ, which was then used for the semi-automatic assessment of mitochondrial morphology. Briefly, following background subtraction, a mitochondrial mask was obtained based on Tom20-positive labeled structures using the Gaussian blur and Auto Threshold (method Default) function. Mitochondrial length and shape were then analyzed via the ‘Skeletonize’ and ‘Particle analyzer’ plugins. Elongation score was calculated as 1/circularity (circularity = 4^∗^pi^∗^(area/perimeter2) where a value of 1 would represent a perfect circle, and hence, fragmented mitochondria.

#### Cytoskeletal analysis

To assess cytoskeletal changes of microglia accepting α-syn aggregates from donor cells we seed 200,000 cells per well onto PDL-coated coverslips and performed the assay as described above (Cell to cell transfer of α-syn aggregates section). After that cells were fixed in 4% PFA, washed 3 times in PBST and stained with Alexa Fluor 647 Phalloidin (1:100, Invitrogen) and DAPI (0.1 μg/mL) for 30 min in PBST. Cells were then mounted and tile images (5x5) were taken using a Zeiss LSM 800 equipped with a 63x oil-objective. Cytoskeletal changes and aggregate count were analyzed using CellProfiler (v3.1.8, Broad Institute of Harvard and MIT, MA, USA) (Kamentsky et al., 2011).

#### Immunohistochemistry

Organotypic slice cultures (OSCs) were fixed for 1 h in 4% PFA followed by 2 washing steps in PBS for 5 min. OSCs were then incubated for 1 h each in PBS containing 15% sucrose and 30% sucrose. After that slices were snap frozen in Optimal Cutting Temperature (OCT) compound (Sakura provided by Thermo Fisher Scientific) and stored at −80°C until they were processed by a cryostat into 40 μm thick sections. Cutted slices were collected using Superfrost ultra plus slides (Thermo Fisher Scientific) and washed 3 times in PTX for 5 min.

Paraffin embedded human brain sections of *post-mortem* Multisystem Atrophy individuals were deparaffinized and treated as follows prior staining. Samples were rehydrated using the following incubation steps: 3x xylene for 5 min, 2x 100% EtOH for 10 min, 2x 95% EtOH for 10 min, 1x 70% for 5 min, 1x 50% EtOH for 5 min, 2x H_2_O dest. For 5 min and 3x PBS for 5 min. After that, antigen unmasking was performed by boiling the samples for 15 min in citrate buffer (10 mM citric acid, 0.05% Tween 20, pH 6.0) followed by three washing steps in PBS and 15 min incubation with proteinase K (0.4 μg/mL) in TE buffer (50 mM Tris base, 1 mM EDTA, 0.5% Triton X-100, pH 8.0) at 37°C.

PFA-fixed human brain sections of *post-mortem* Dementia with Lewy Bodies individuals were washed three times with PBS for 15 min prior staining. Blocking solution containing PTX and 5% normal goat serum (Vector Laboratories, Burlingame, CA, USA) was applied for 30 min. The primary antibody rat anti-CD11b (1:250; Serotec by Bio-Rad) and mouse anti-α-synuclein (1:100; BioLegend) were applied overnight at 4°C. After three washing steps in PTX, the secondary antibodies goat anti-rat-AlexaFluor488 (1:250; Invitrogen) and goat anti-mouse-AlexaFluor594 (1:250; Invitrogen) were applied for 2 h. DAPI was used for nuclear counterstaining at 0.1 mg/mL for 30 min in PBS. Images were taken using a Zeiss LSM 800 microscope.

#### Cranial window placement and intracranial injection of α-synuclein

Cranial window installation and *in vivo* 2-photon image acquisition were carrying out as previously described elsewhere (Hefendehl et al., 2012; Holtmaat et al., 2009). Briefly, 7-month-old male Cx3cr1^eGFP^ mice received an intraperitoneal injection (i.p.) of 1.5 mg/kg ketamine and 0.1 mg/kg xylazine, followed by subcutaneous injections (s.c.) of 0.1 mg/kg buprenorphine, 6 mg/kg dexamethasone, and 7 mg/kg cefotaxime. Bepanthen eye ointment was applied to the eyes to avoid drying. Surgical instruments were sterilized in a bead heater (GerminatorTM 500; CellPoint Scientific Inc. Gaithersburg, MD, USA) and hairs on top of the head were removed. The mouse was then put into a stereotactic frame and the disinfected skin was then removed using sharp scissors. The periosteum was removed by gently scraping with a scalpel to increase the gluing capacity between the ring and the skull. Two small holes were drilled into the skull using Schick driller C1 device (Schick GmbH, Schemmerhofen, Germany). Thereafter the mouse received a stereotaxic injection of 0.5 μL 0.1 μM α-syn-Atto550 with a speed of 0.1 μl/min for each hole in the cortex. After the injection, the needle was kept in place for an additional 10 min before it was slowly withdrawn to avoid reflux up the needle tract. Right after, 4 mm diameter craniotomy was performed over the right hemisphere and the craniotomy was rinsed with physiological saline solution. Subsequently, using UV activated dental cement (Venus flow syringe assortment, MW dental) a 5-mm coverslip was placed on the top of the cranial window. A custom-made titanium ring was glued on the skull with the help of Pattex super glue gel. After the operation, the mouse was put under infrared light for recovery. The body temperature was controlled throughout the procedure and maintained at 37°C. After a short recovery period, 2-photon imaging was performed.

#### *In vivo* 2-photon imaging

A Ti:Sapphire 2-photon laser scanning microscope was used with a Nikon water-immersion objective (25x, 1,10 NA) and Nikon NIS Elements AR 4.20.03 (Build 995; Düsseldorf, Germany). Imaging was performed under isoflurane anesthesia (1.5%, flow ∼800 mL/min). The mouse was put onto a heating blanket and rectal temperature was kept constant at 37°C. All images were taken using 920 nm wavelength for EGFP and RFP. An overview stack (x, y, z: 522 × 522 × 75 μm; 1 μm z-step size; pixel size, 1.02 μm/pixel) was taken for orientation, before areas of interest were randomly chosen. Z stacks for each region of interest (x, y, z: 155 × 155 × 70 μm) with a pixel size of 0.3 μm/pixel and a z-spacing of 0.5 μm were acquired. For time-lapse recordings, images were taken every 30 s.

#### Microscopy and image analysis

All experiments were examined with a Nikon Eclipse Ti fluorescence microscope (Nikon, Tokyo, Japan), a Zeiss laser scan microscope 800 (Carl Zeiss, Oberkochen, Germany) or a Ti-Sapphire 2-photon laser scanning microscope (Nikon). Acquired images were processed using NIS-elements 4 (Nikon) and Fiji ImageJ (Wayne Rusband, National Institute of Health, USA). Three-dimensional reconstructions were processed using Imaris – Microscope Image Analysis Software (Bitplane, Oxford Instruments plc, Abingdon, UK).

### Quantification and statistical analysis

Data were evaluated using Graph Pad Prism and presented as mean ± SEM of at least three independent experiments with three replicates. Data were analyzed for Gaussian distribution. When data passed the normality test statistical comparisons of vehicle controls versus treatment were performed with one-way ANOVA or two-way ANOVA followed by a Tukey’s test. Otherwise, data were analyzed with the Kruskal-Wallis test and a Dunn’s post hoc test for non-parametric data. Levels of significance are indicated as ^∗^p < 0.05; ^∗∗^p < 0.01; ^∗∗∗^p < 0.001; ^∗∗∗∗^p < 0.0001. Statistical details for all experiments can be found in the respective figure legends.

## Data Availability

RNA-seq data have been deposited at GEO (Database: GSE152100, GSE166127). Accession numbers are listed in the key resource table. Any additional data reported in this paper will be shared by the lead contact upon request. This paper does not report original codes.

## Supplemental information

Table S1. DE gene analysis of transcriptomic changes in donors (0 vs. 300 min). Genes whose expression increases or decreases in donors after 0 and 300 min of co-culture with acceptors, related to Figure 1D
