# Supplementary material for: Microglia jointly degrade fibrillar alpha-synuclein cargo by distribution through tunneling nanotubes
Source: Cell. 2021 Sep 30;184(20):5089–5106.e21. doi: 10.1016/j.cell.2021.09.007 (PMC8527836; doi:10.1016/j.cell.2021.09.007)
Supplement: Table S1. DE gene analysis of transcriptomic changes in donors (0 vs. 300 min). Genes whose expression increases or decreases in donors after 0 and 300 min of co-culture with acceptors, related to Figure 1D [file mmc1.pdf]

Cell, Volume *184*

Supplemental information

Microglia jointly degrade fibrillar

alpha-synuclein cargo by distribution

through tunneling nanotubes

Hannah Scheiblich, Cira Dansokho, Dilek Mercan, Susanne V. Schmidt, Luc Bousset, Lena Wischhof, Frederik Eikens, Alexandru Odainic, Jasper Spitzer, Angelika Griep, Stephanie Schwartz, Daniele Bano, Eicke Latz, Ronald Melki, and Michael T. Heneka

Supplementary Table 1
DE gene analysis of transcriptomic changes in donors (0 vs. 300 min)
Genes whose expression increases or decreases in donors after 0 and 300 min of co-culture with acceptors. Related to Figure 1D.

	Mean expression			
**Gene name**	**Donors 0min**	**Donors 300min**	**FC (300min vs 0min)**	**step-up Value (300min vs 0min)**
Klf2	2102,6	262,3	-8,0	7,72E-03
Serpine1	181,7	31,7	-5,7	7,42E-05
Fbxo32	291,3	72,5	-4,0	1,55E-04
Gm20498	18,7	5,1	-3,7	3,36E-02
Klf4	504,4	148,4	-3,4	1,48E-04
Bhlhe40	262,8	78,6	-3,3	1,51E-02
Cxcr4	2748,2	890,6	-3,1	9,05E-05
Wee1	179,3	58,6	-3,1	1,48E-04
Plk3	149,4	49,3	-3,0	4,91E-03
Dusp1	5056,4	1837,2	-2,8	2,10E-02
Tsc22d3	1137,5	468,9	-2,4	1,48E-04
Gadd45a	272,2	112,4	-2,4	1,21E-02
Plaur	478,5	206,3	-2,3	4,21E-02
Chac1	226,2	97,5	-2,3	6,00E-03
Il17ra	542,0	234,6	-2,3	1,08E-02
Ercc6	276,1	124,4	-2,2	1,19E-02
Klf11	91,1	42,6	-2,1	3,28E-02
Adgre5	1052,3	512,4	-2,1	2,85E-07
Zfp568	84,5	42,2	-2,0	5,75E-03
Tcp11l2	335,8	167,6	-2,0	2,57E-02
Sgms1	852,5	429,0	-2,0	7,72E-03
Ifrd1	2804,8	1449,5	-1,9	4,12E-02
Zfp747	164,3	84,9	-1,9	4,10E-03
Sfn	283,2	146,8	-1,9	4,94E-03
Eno2	182,8	95,5	-1,9	7,51E-03
Klf10	534,8	280,3	-1,9	4,10E-03
Ankrd28	179,0	95,6	-1,9	4,79E-02
Map2k3	2178,3	1180,6	-1,8	7,29E-03
Mical1	702,5	381,0	-1,8	4,10E-03
Mindy1	1540,7	851,0	-1,8	1,87E-02
Tgif1	1879,8	1056,2	-1,8	4,65E-03
Fam20c	1479,2	846,3	-1,7	3,96E-02
Ell	451,6	259,9	-1,7	4,94E-03
Trim47	975,8	575,4	-1,7	4,16E-02
Rassf8	366,7	217,2	-1,7	2,95E-02
Cd44	5386,3	3191,4	-1,7	1,66E-04
Lhfpl2	3265,1	1947,1	-1,7	1,58E-04
Gramd1b	1444,3	872,4	-1,7	2,71E-02
Baiap2	658,8	404,9	-1,6	2,90E-02
Lmna	3724,0	2290,6	-1,6	4,54E-02
Synj2	254,6	159,5	-1,6	5,57E-03
Mtmr10	388,9	243,7	-1,6	1,08E-02
Mdm2	4525,9	2888,6	-1,6	4,04E-02
Emp1	2380,6	1522,1	-1,6	4,62E-03
1700017B05Rik	818,8	524,6	-1,6	9,33E-03
Fabp4	1044,0	683,3	-1,5	3,91E-02
Ivns1abp	526,2	346,6	-1,5	4,42E-02
Pip5k1c	1501,8	990,9	-1,5	2,41E-02
Emsy	325,2	217,3	-1,5	1,24E-02
Hnrnph1	1524,1	1025,5	-1,5	2,10E-02
Furin	1366,3	921,6	-1,5	4,04E-02
Dmxl2	294,0	200,3	-1,5	2,71E-02
Tmbim1	1236,9	849,7	-1,5	2,18E-02
Bbc3	1088,9	761,6	-1,4	2,71E-02
Gpr137b	2661,7	1902,8	-1,4	4,42E-02
Ski	1066,3	765,8	-1,4	1,58E-02
Slc27a1	1232,8	896,0	-1,4	8,68E-03
Ctnnb1	6213,8	4652,5	-1,3	4,42E-02

Arfgap3	304,5	229,7	-1,3	4,80E-02
Arl8b	2205,8	1687,8	-1,3	1,73E-02
Ewsr1	1660,7	1309,3	-1,3	8,44E-03
Rap2b	2574,3	3144,0	1,2	4,19E-02
Serf2	9003,9	11139,4	1,2	3,91E-02
Timm10b	1237,3	1570,2	1,3	4,62E-03
Rap1gds1	779,7	1044,4	1,3	1,63E-02
Limd2	4147,9	5908,0	1,4	3,38E-02
Vapb	1376,9	1968,8	1,4	1,51E-02
Dgkh	465,5	674,7	1,4	4,04E-02
Snx6	701,1	1039,5	1,5	4,10E-03
Mtln	255,8	389,5	1,5	8,44E-03
Enox2	184,8	286,2	1,5	2,13E-02
Fbxw4	351,9	576,2	1,6	3,36E-02
Fnbp1l	627,8	1104,1	1,8	1,12E-02
3830406C13Rik	196,8	352,6	1,8	8,13E-04
Mtg1	67,1	121,3	1,8	4,91E-02
Maf	3533,0	6431,1	1,8	4,94E-03
F630028O10Rik	358,4	732,7	2,0	4,54E-02
Fam72a	27,8	97,2	3,5	2,57E-02
